# Structural abnormalities in islets from very young children with cystic fibrosis may contribute to cystic fibrosis-related diabetes

**DOI:** 10.1038/s41598-017-17404-z

**Published:** 2017-12-08

**Authors:** Marika Bogdani, Scott M. Blackman, Cecilia Ridaura, Jean-Pierre Bellocq, Alvin C. Powers, Lydia Aguilar-Bryan

**Affiliations:** 1 Benaroya Research Institute, Seattle, WA USA; 2 Division of Pediatric Endocrinology, Johns Hopkins University School of Medicine, Baltimore, MD USA; 3 Department of Pathology, Instituto Nacional de Pediatría, Mexico City, Mexico; 4 Department of Pathology, Hôpital de Hautepierre, Strasbourg, France; 5 Division of Diabetes, Endocrinology, and Metabolism, Department of Medicine, and Department of Molecular Physiology and Biophysics, Vanderbilt University School of Medicine, and VA Tennessee Valley Healthcare System, Nashville, TN USA; 6 Pacific Northwest Diabetes Research Institute, Seattle, WA USA

## Abstract

Cystic fibrosis (CF)-related diabetes (CFRD) is thought to result from beta-cell injury due in part to pancreas exocrine damage and lipofibrosis. CFRD pancreata exhibit reduced islet density and altered cellular composition. To investigate a possible etiology, we tested the hypothesis that such changes are present in CF pancreata before the development of lipofibrosis. We evaluated pancreas and islet morphology in tissues from very young CF children (<4 years of age), and adult patients with CF and CFRD. The relative number of beta-cells in young CF tissues was reduced by 50% or more when compared to age-matched controls. Furthermore, young CF tissues displayed significantly smaller insulin-positive areas, lower proportion of beta-cells positive for the proliferation marker Ki67 or the ductal marker CK19 vs. control subjects, and islet inflammatory cell infiltrates, independently of the severity of the exocrine lesion and in the absence of amyloid deposits. CFRD pancreata exhibited greater islet injury with further reduction in islet density, decreased relative beta-cell number, and presence of amyloid deposits. Together, these results strongly suggest that an early deficiency in beta-cell number in infants with CF may contribute to the development of glucose intolerance in the CF pediatric population, and to CFRD, later in life.

## Introduction

Cystic fibrosis (CF) is the most common lethal genetic disorder of childhood in the Caucasian population and is due to recessive mutations in *ABCC7*, the gene encoding the CF transmembrane conductance regulator (CFTR). When CFTR is functionally compromised, abnormal exit of Cl^−^ from epithelial cells defines a progressive multi-organ disease, affecting the lungs, exocrine pancreas (insufficiency), and gut pathology^1^. With the development of new therapies and extended life expectancy CF patients represent the population with the highest risk for age-related diabetes mellitus (CFRD), currently affecting ~20% of adolescents and ~50% of young adults^2–4^.

The etiology of CFRD is complex, but its causes and pathophysiology remain largely unknown. Currently, glucose intolerance and diabetes in CF are thought to be mainly related to insulin deficiency due to reduced beta-cell mass, function, or a combination of both^5–10^. The observation of pancreatic islets amidst a lipoatrophic or fibrotic tissue formed the basis for the view that pancreatic fibrosis resulting from chronic pancreatic pathology is the main cause of beta-cell dysfunction and destruction in CF. Nevertheless, the progressive exocrine damage alone cannot entirely explain the development of CFRD; not all CF patients with exocrine pancreatic insufficiency or severe pancreatic fibrosis develop CFRD^2,3^. In addition, although pancreatic insufficiency presents within the first few months of life, diabetes develops years after exocrine pancreatic destruction has taken place^2,11^. Because the anatomic component of pancreatic insufficiency/fibrosis per se cannot adequately explain CFRD, we hypothesized that beta-cell mass may be reduced in the CF pancreas prior to development of fibrosis.

To test this hypothesis, we studied pancreatic tissues from a cohort of children (<4 years of age) who died of CF. We show for the first time that beta-cell mass is reduced in pancreata from these children compared to age-matched controls without CF, independently of the severity of exocrine damage. We found that young CF islets display evidence for a reduced rate of beta-cell replication and neogenesis, leukocyte infiltration, and absence of amyloid deposits. The presence of a smaller-than-normal beta-cell population with lower capacity to generate new beta cells could provide the morphologic basis for the altered glucose tolerance observed in young patients with CF and the development of CFRD later in life.

## Results

### Exocrine fibrotic or fatty transformation is not a feature of the young CF tissues

To determine the relationship between islet morphological changes and severity of exocrine pathology in CF, we first evaluated histologic changes in the exocrine pancreas of CF and CFRD patients. These results are summarized in Supplementary Table S1 and representative images are shown in Supplementary Fig. S1. The degree of exocrine damage varied among patients younger than 1 year of age and did not correlate with the patient’s age (Supplementary Fig. S2). This lack of correlation was most evident in the tissues from patients 6 month old and younger (Supplementary Fig. S2). Greater destruction of the acinar tissue with age was noticeable in tissues from young CF patients 1 to 4 years of age, with 75% showing severe exocrine damage (grade 4 or 5) as compared to tissues from younger (<1 year) patients, of which only 23% were grade 4 (Supplementary Table S1). These data provide support for the absence of exocrine lipofibrotic replacement and cystic dilation of the pancreatic ducts in the very young CF pancreas.

### Altered islet size is a feature of the CF pancreas with advanced exocrine damage

**Figure 1:**
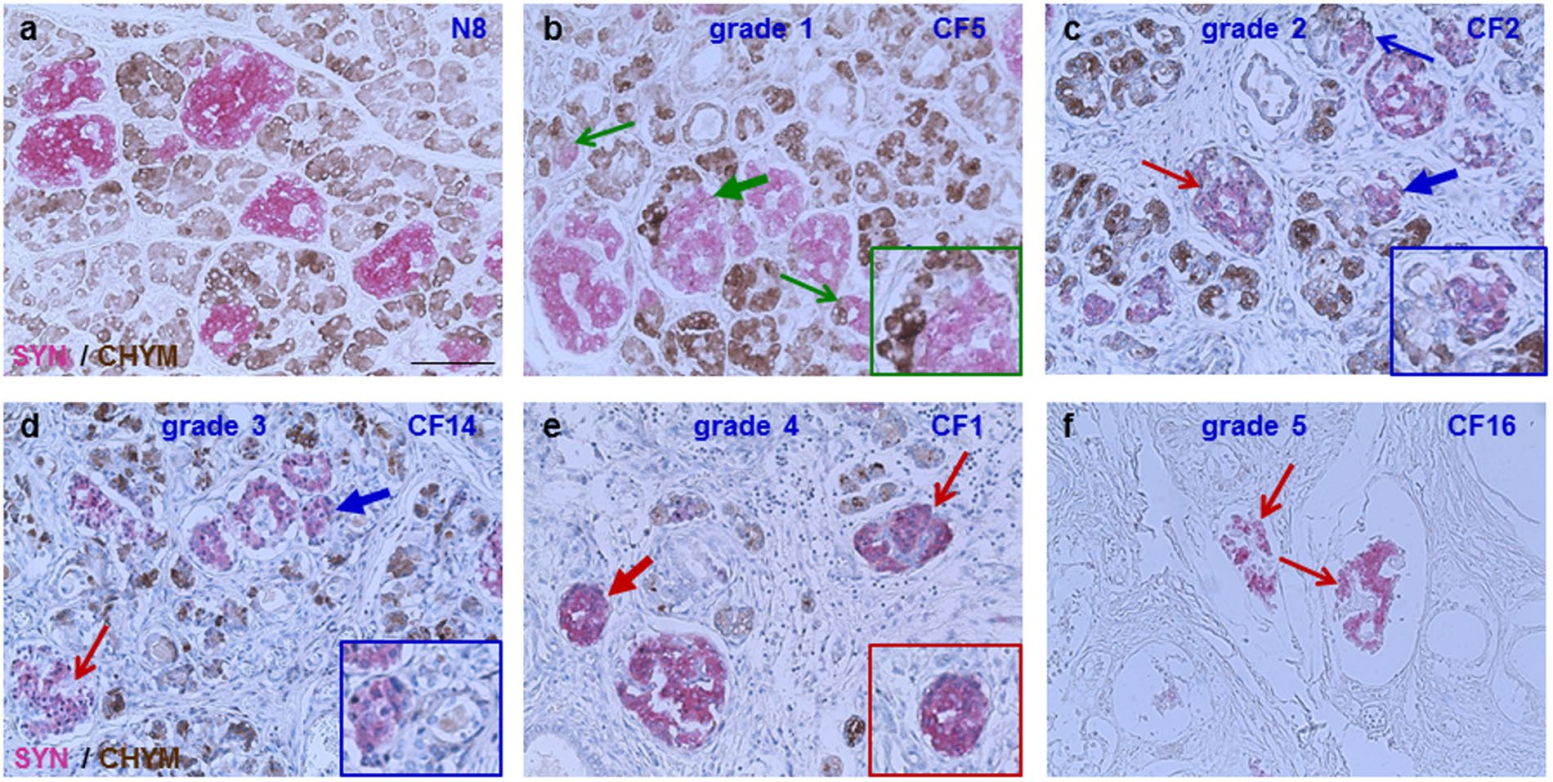
Islet architecture in the young CF pancreas. Immunostaining for chymotrypsin (CHYM, brown) and synaptophysin (SYN, magenta) in normal (**a**) and CF (**b**–**f**) pancreas. The CF tissues from very young patients show different degrees of histopathological changes in the exocrine pancreas. SYN-positive cells are located adjacent to CHYM-positive acini, CHYM-negative duct or duct-like structures, or surrounded by fibrosis, and are indicated by the green, blue, or red arrows and insets, respectively. The islets indicated by the thicker arrows are shown magnified within the insets. Scale bar: 50 µm.

To determine whether islets from young CF patients show morphological characteristics different from those of adult CF and CFRD patients, we examined the tissues of younger patients separately and according to their histopathologic grade. For grades 1 or 2 young CF pancreas tissues, islet cell number was comparable to that observed in the age-matched normal tissues (Fig. 1a–c, Supplementary Fig. S3). In these CF tissues, islets were of variable size (Table 1), and occurred as single units or in small groups, arranged in a back-to-back position. The endocrine cells also occurred as dispersed single cells or clusters, adjacent to exocrine acini, duct-like structures, or pancreatic ducts (Fig. 1b and c, Supplementary Fig. S3).

**Table 1:** Quantification of the number of endocrine cells per islet.

Tissues	N	Number of cells per islet^a^	
		(mean ± SD)	range
Young control	11	69 ± 25	23 to 300
Young CF	16	61 ± 23	22 to 280
**Histopathologic grade**			
1 and 2	6	75 ± 20	22 to 280
3	5	57 ± 18	22 to 160
4	3	46 ± 21	21 to 160
4	1 (CF13)	35 ± 18^b,c^	25 to 80
5	1 (CF16)	33 ± 16^b,c^	21 to 70

^a^Number of cells per islets represents the number of nucleated synaptophysin-positive cells counted within the cross-sections of the pancreatic islets. Data are mean ± SD of measurements obtained from the indicated number of cases. ^b^Represents the mean ± SD of measurements obtained from 310 and 180 islets counted in the CF13 and CF16 cases, respectively. ^c^ *p* < 0.01, vs. controls.

For grades 3 and 4, islets were smaller than those in pancreata with less exocrine damage (Table 1). A substantial decrease (50–70%) in the chymotrypsin-positive areas was observed, therefore, islets and endocrine cell clusters were located mainly in midst of fibrosis or in vicinity of the duct-like structures (Fig. 1d and e, Supplementary Fig. S3). In the CF13 pancreas (Supplementary Fig. S3), the islets were significantly smaller, and the endocrine cells clusters were numerous, containing three to ten synaptophysin-positive (SYN) cells. The most prominent changes in islet morphology were observed in grade 5 CF pancreas (CF16), where islets were surrounded by thick collagen bundles and intra-islet fibrosis (Fig. 1f).

In the adult CF and CFRD pancreas tissues, islets were either surrounded by fibrosis or embedded in adipose tissue, consistent with the previously described “fibrotic” or “lipoatrophic” patterns^6,8,9^. In the CF tissues showing the “fibrotic” pattern (CF17, CF18, CFRD2), islets occurred mainly in clusters and appeared as masses of densely packed endocrine cells (Supplementary Fig. S4). These tissues contained 250, 180, and 50 islets per cm^2^ tissue, respectively. In CF19 and other CFRD pancreata with the “lipoatrophic” pattern, islets were scarce (7 to 21 islets per cm^2^ tissue), surrounded by adipose tissue (Supplementary Fig. S4), and showed altered morphology.

Taken together, these results suggest that changes in islet architecture are more evident in the CF tissues in advanced stages of exocrine destruction. In the adult CF pancreas, altered islet morphology and density were more pronounced in the presence of CFRD and in tissues with a “lipoatrophic” pattern.

### Insulin-positive (INS) areas are decreased in the young CF pancreas with minimal exocrine pancreas damage

**Figure 2:**
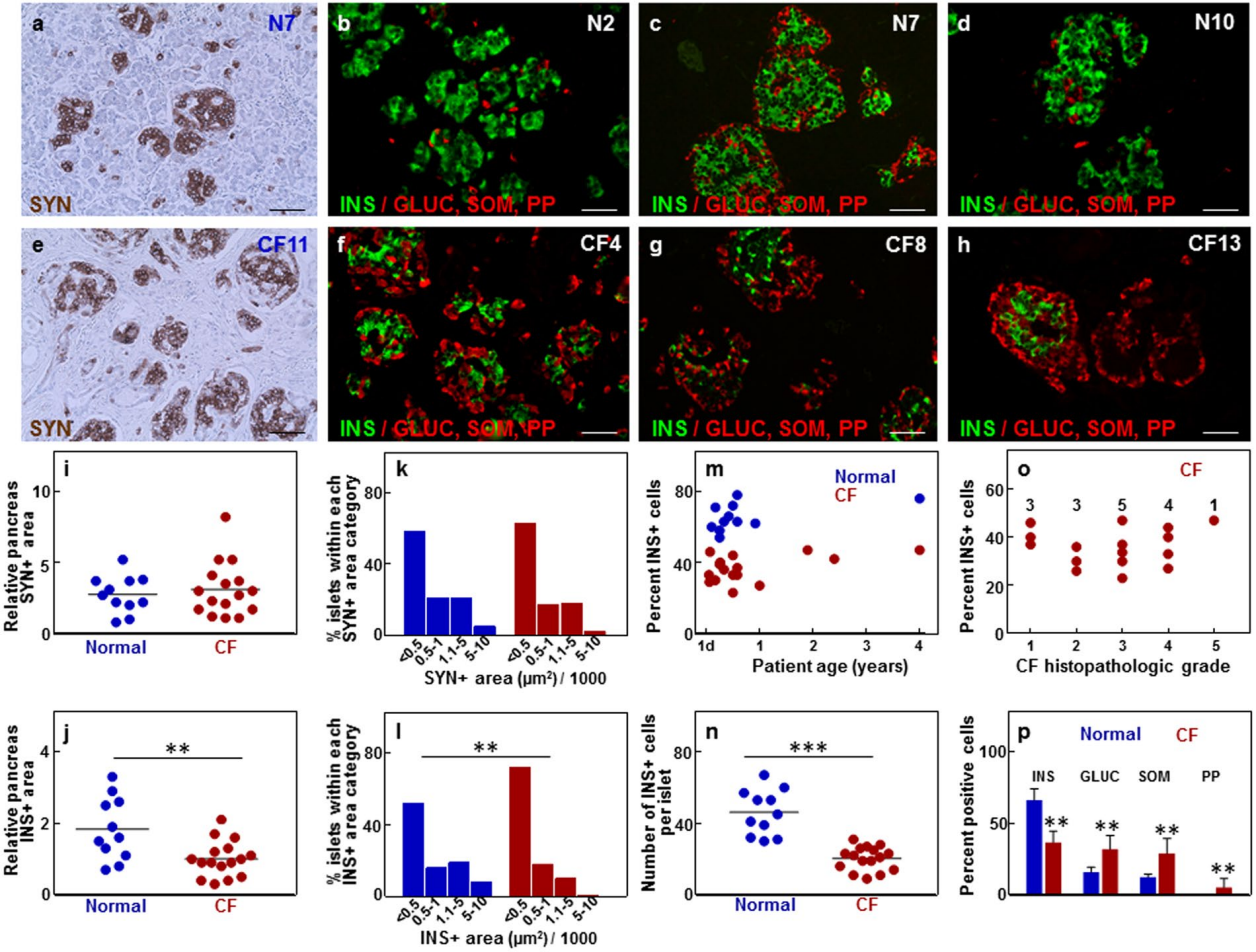
Quantitative analysis of the relative endocrine cell areas and islet cell composition. Immunostaining for synaptophysin (SYN) in brown, insulin (INS) in green, and glucagon (GLUC), somatostatin (SOM) and pancreatic polypeptide (PP) in red in normal (**a**–**d**) and CF (**e**–**h**) pancreas. Scale bars: 50 µm. (**i** and **j**) Plots of relative pancreatic SYN-positive (SYN+) and INS-positive (INS+) areas. **p < 0.001 vs. normal tissues. (**k** and **l**) Histograms of the distribution of the SYN+ and INS+ areas in the young normal and CF pancreas tissues indicate percent islets with SYN+ or INS+ areas falling within each islet SYN+ or INS+ area category. Data include measurements of 18622, 7004, and 6597 islets with SYN− or INS-positive areas <500 µm^2^, 500–1000 µm^2^, or >1000 µm^2^, respectively, from 16 CF and 11 normal pancreas tissues. **p < 0.001 vs. normal tissues. (**m**) Plots of percent islet INS+ cells in the young CF pancreas as function of age. (**n**) Number of INS+ cells per islet in the young normal and CF pancreas. ***p < 0.0001 vs. normal tissues. (**o**) Correlation analysis of percent islet INS+ cells with the CF histopathological grade. The numbers of CF tissues falling into each grade category are shown above the measurements. Each dot represents one young CF pancreas (n = 16). The correlation coefficient is R = 0.06. (**p**) Quantitative analysis of islet cellular composition. Data are mean ±SD of measurements obtained from young normal (n = 11) and young CF (n = 16) pancreata. **p < 0.001 vs. age-matched controls. Red circles and bars, young CF tissues; blue circles and bars, young normal tissues.

As shown in Fig. 2a,e and i, Table 1, and Supplementary Table S1, the islet endocrine areas and cell number in young CF tissues were not different from that of controls. Whether comparable relative islet areas reflect comparable sizes of the total islet cell population in the CF and normal pancreas could not be determined since the pancreas weights were not available. However, referring to earlier studies that reported similar pancreas weights in normal and CF infants up to 6 months of age^12,13^, it appears that, at least in the first 6 months of life, the endocrine cell mass is comparable between the normal and CF tissues.

In adult CF tissues (CF 17*–*19, CFRD 1*–*8), the relative synaptophysin-positive (SYN) islet areas were also comparable to those in age-matched controls (1 ± 2% and 1 ± 1%, respectively, *p* > 0.05). When the CF tissues were examined according to the presence or absence of CFRD, smaller relative SYN areas were found in the CFRD (0.3 ± 0.6%) vs. CF tissues (1 ± 1%). Examination of the CF tissues according to the histologic pattern of the exocrine lesion indicated that the relative islet areas were smaller in the CF tissues with “lipoatrophic” pattern (0.1 ± 0.1%, n = 8) as compared to 4.5 ± 3.7% (n = 3, *p* < 0.05) observed in those with the “fibrotic” pattern. A similar trend was found when the number of cells per islets was examined in the CF and CFRD tissues. CFRD islets contained fewer SYN cells (32 ± 12 cells per islet) than the CF islets (59 ± 12 cells per islets, *p* < 0.05). Similarly, islets in the CF tissues with the “lipoatrophic” histopathological pattern contained on average fewer cells than those presenting the “fibrotic” pattern (35 vs. 54 cells per islet, respectively).

The data indicate that islet size is not affected in the young CF tissues, and that CFRD and the “lipoatrophic” histopathological pattern are associated with decreased relative pancreatic islet area and reduced islet endocrine cell number.

Despite showing an islet size comparable to that of control tissues, the young CF tissues exhibited significantly lower relative pancreas INS areas, reduced islet INS areas, reduced number of INS cells per islet, and a smaller percentage of INS cells, compared to age-matched controls (Fig. 2f–h,j–n). Correlation analysis between relative INS cell number and histopathological grade (Fig. 2o) did not indicate that a lower beta-cell percentage in the young CF tissues related to more extensive exocrine loss and tissue fibrosis. The glucagon-positive (GLUC) and somatostatin-positive (SOM) cells accounted for the majority (61 ± 3%) of the endocrine cells and occurred in similar proportions (Fig. 2p, Supplementary Fig. S5). This contrasts with their distribution in normal islets where these endocrine cell types accounted for 28 ± 3% of the endocrine cells. The pancreatic polypeptide-positive (PP) cells were more frequently observed in the CF pancreas (3 ± 2%) vs. controls (<0.1% of the endocrine cells, Fig. 2p).

Adult CF and CFRD tissues were also characterized by a significantly lower relative proportion of INS cells vs. controls (Supplementary Fig. S6), as previously reported^6^. Regarding islet cellular composition, no distinction could be made between CF and CFRD tissues or between CF and CFRD tissues with the “fibrotic” or “lipoatrophic” patterns.

The data indicate that islet cellular composition is altered in the young CF pancreas, likely due to a reduced number of beta cells, and that these changes persist in the adult CF and CFRD pancreas.

### Beta-cell proliferation and neogenesis are reduced in the CF pancreas

**Figure 3:**
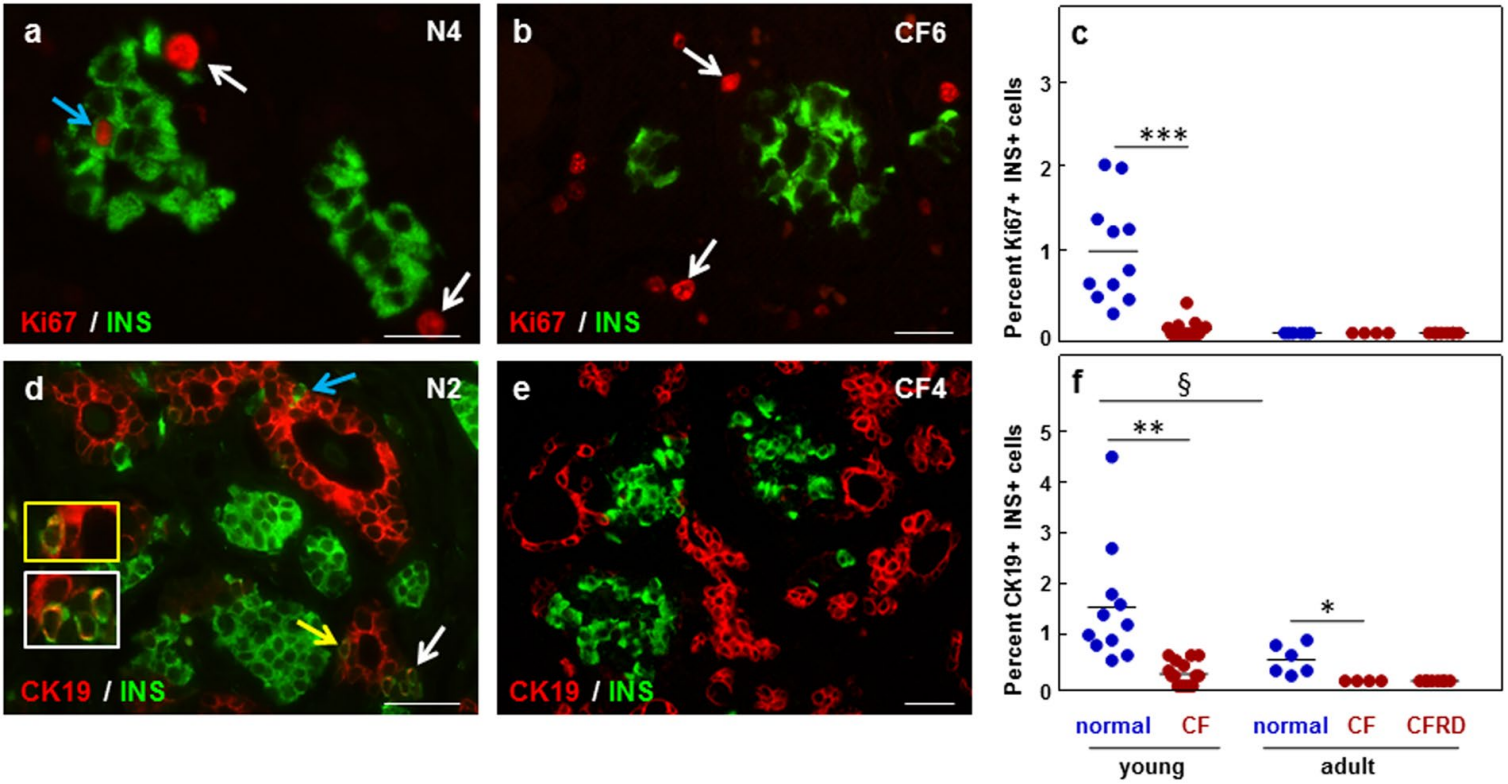
Pancreatic beta-cell proliferation and neogenesis in normal and CF pancreas. (**a** and **b**) Co-labeling for Ki67 (red) and insulin (INS, green) in normal (**a**) and CF (**b**) pancreas. Light blue arrow points to a Ki67+ INS+ cell; white arrows, Ki67+ INS− cells in the extra-islet tissue. Higher magnification of the Ki67+ INS+ cell indicated by the magenta or light blue arrows is shown in the inset of the same color. (**c**) Quantification of proliferating INS− positive cells in normal and CF pancreatic tissues. (**d** and **e**) Double immunohistochemistry for the human pancreatic duct cell marker cytokeratin 19 (CK19, red) and INS (green) in normal and CF pancreas. Arrows point to CK19+ INS− cells. Yellow and white arrows point to CK19+ INS+ cells. Higher magnification of the INS+ CK19+ positive cells indicated by the white and yellow arrows is shown in the insets of the same color. (**f**) Quantification of cells co-labeled for CK19 and INS. Data were obtained from young normal (n = 11), adult normal (n = 6), young CF (n = 16), adult CF (n = 3), and CFRD (n = 8) pancreata. *p < 0.05, ^§^ *p* < 0.05, **p < 0.001, ***p < 0.0001. Asterisks, significance vs. age-matched controls; section sign, significance vs. young controls. Blue circles, normal pancreas; red circles, CF pancreas. Scale bars: 25 μm (**a** and **b**), 50 μm (**d** and **e**).

To determine if pancreatic beta-cell growth is affected in CF, we examined the rate of beta-cell proliferation and neogenesis in CF and CFRD pancreata. In contrast to normal tissues where proliferating beta-cells were regularly observed, few cells positive for Ki67 and insulin occurred in the young CF tissues, accounting for <0.01% of INS cells (Fig. 3a–c). No proliferating beta cells were observed in adult CF tissues (Fig. 3c). No proliferating GLUC, SOM, or PP cells were detected in any control or CF tissues.

We further determined whether new endocrine cells possibly generated through neogenesis from ductal cells were present in CF pancreata. Cells co-labeled for insulin and CK19 were observed in the young control tissues, adjacent to the CK19-positive borders of the ducts and duct-like structures (Fig. 3d), and accounted for 1.3 ± 1.2% of the INS cells. Insulin and CK19 double-positive cells occurred in similar sites in the young CF pancreas tissues, at significantly lower occurrence (Fig. 3e,f). CK19 and INS double-positive cells were also present in normal adult pancreata, in line with previous work^14^, but were virtually absent in the adult CF and CFRD tissues (Fig. 3f). Cells co-labelled for glucagon and CK19 were observed only in two neonatal control tissues (N1 and N2), and counted for less than 1% of GLUC cells. No glucagon and CK19-double-positive cells were detected in any of the other controls or CF cases (Supplementary Fig. S7). The data indicate that both beta-cell proliferation and neogenesis were significantly altered in the young CF pancreas and these alterations persisted in the adult CF pancreas.

### Young CF islets are infiltrated by dispersed inflammatory cells

**Figure 4:**
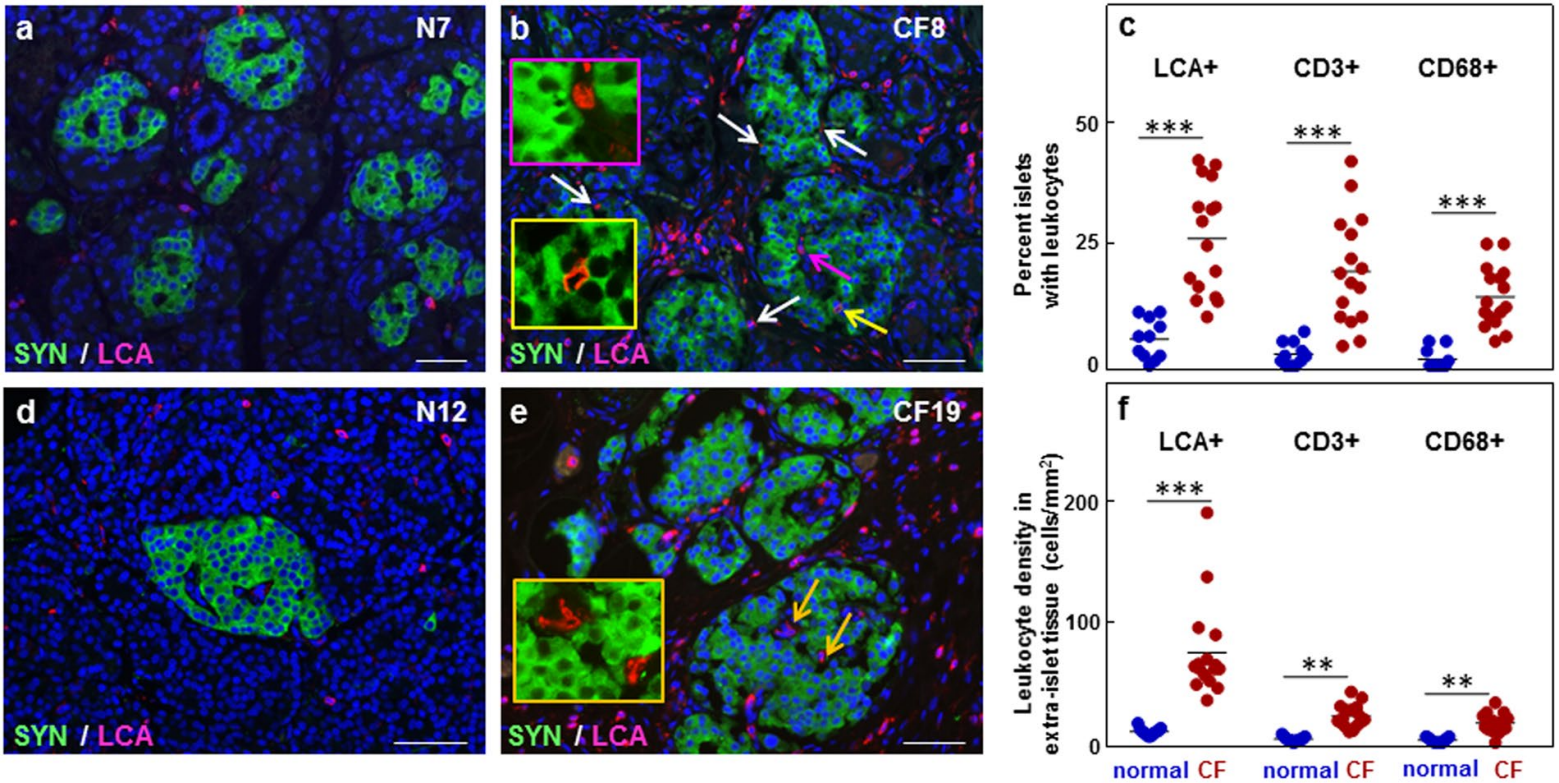
Inflammatory cell infiltrates in islets and extra-islet tissue in the normal and CF pancreas. (**a** and **b**) Immunostaining for synaptophysin (SYN, green) and the leukocyte common antigen (LCA, magenta) in young normal (**a**) and CF (**b**) pancreas. (**c**) Quantification of LCA−, CD3−, and CD68− positive cells associated with pancreatic islets. (**d** and **e**) Immunostaining for SYN (green) and LCA (magenta) of adult normal (**d**) and CF (**e**) pancreas. Arrows point to inflammatory cells in contact with islet cells. Higher magnification of the LCA+ cells indicated by the yellow and magenta arrows in (**b**), or orange arrows in (**e**) is shown within the inset of the same color. Scale bars: 50 µm. (**f**) Quantification of LCA−, CD3−, and CD68− positive cells in the extra-islet tissue. Blue circles, young normal pancreas; red circles, young CF pancreas. Data are obtained from young normal (n = 11), and young CF (n = 16) pancreata. **p < 0.001, ***p < 0.0001 vs. normal tissues.

In young control tissues, LCA inflammatory cells were scarce, while frequently observed around or within islets in young CF tissues (Fig. 4a,b). The proportion of islets with LCA cells and the LCA cell density per islet in the young CF tissues were significantly higher compared to controls (Fig. 4c, Supplementary Fig. S8). No correlation was observed between the islet INS areas or islet INS+ cell number and the proportion of islets with inflammatory cell infiltrates (Supplementary Fig. S8). Adult CF and CFRD tissues had fewer LCA cells associated with the islets compared to younger CF tissues (Fig. 4d,e, Supplementary Fig. S8). When tissues were examined according to the histopathologic pattern, a significantly larger proportion of islets in CF and CFRD tissues displaying the “fibrotic” pattern contained immune cells compared to controls (Supplementary Fig. S8). Both CD68 monocytes/macrophages and CD3 lymphocytes were present in young CF islets (Fig. 4c, Supplementary Fig. S8). CD3-positive cells were also present in adult CF islets, while no CD68-positive cells were observed in these islets, although CD68-positive leukocytes were present in the surrounding extra-islet tissue (Supplementary Fig. S8).

LCA inflammatory cells were also present in the extra-islet compartment. Numerous LCA cells occurred in the young CF pancreata, while their number was significantly lower in the adult CF and CFRD samples (Fig. 4f). In the latter, the presence of LCA cells was not affected by CFRD (*p* > 0.05). However, their tissue density varied with the CF histopathological pattern. The ‘fibrotic’ adult pancreas tissues contained more LCA cells than those with the ‘lipoatrophic’ pattern (36 ± 11 cells/mm^2^ vs. 9 ± 1 cell/mm^2^, *p* < 0.001). The vast majority of the LCA inflammatory cells stained for CD3 or CD68 (Supplementary Fig. S8).

The data indicate that in the young CF tissues, islets are infiltrated by inflammatory cells. The extent of infiltration is attenuated in the adult CF and CFRD pancreas, in spite of the presence of ongoing chronic pancreatitis.

### Amyloid formation is not characteristic for young CF islets

**Figure 5:**
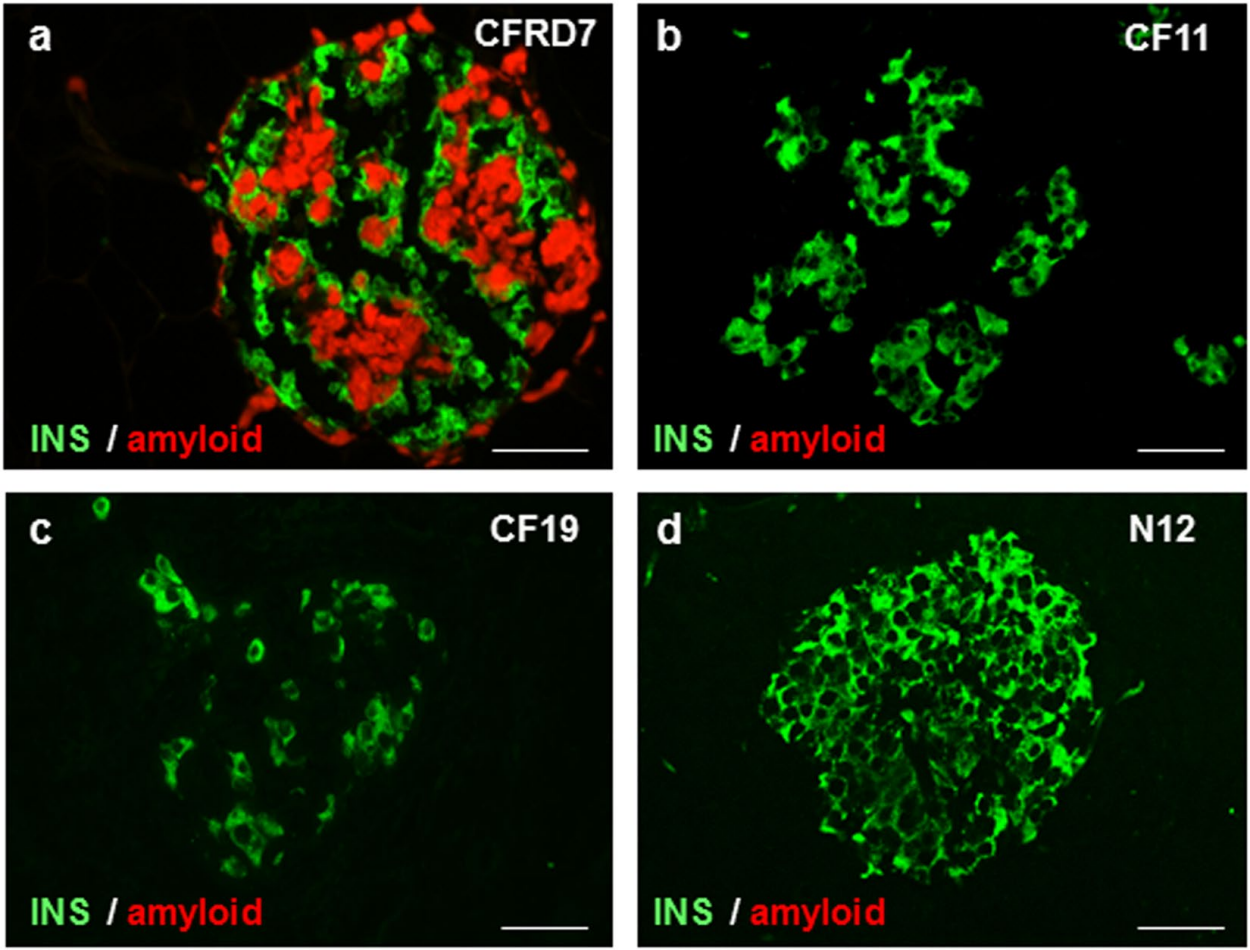
Amyloid deposits in the islets of patients with CFRD. (**a**–**d**) Staining for insulin (INS, green) and thioflavin S staining (red) for amyloid in CFRD (**a**), young CF (**b**), adult CF (**c**), and normal (**d**) pancreas. Scale bars: 50 µm.

Islet amyloidosis has been reported in a subset of patients with CFRD^7,15^. To determine whether the lower beta-cell number we observed in very young CF pancreata is associated with amyloid formation, pancreas sections were stained for insulin and amyloid. Adult CF and CFRD tissues were evaluated concomitantly. We observed amyloid deposits in islets from three of eight patients with CFRD, but in none of the other CFRD, young and adult CF, or control tissues (Fig. 5a–d). Apoptotic TUNEL-positive beta-cells were detected in two of the three cases with amyloid, but not in other CF or control cases (Supplementary Fig. 9). Enhanced beta-cell loss in CF islets with amyloid suggests that changes in the islet microenvironment can lead to beta-cell damage. Apoptotic cells were scarce in the extra-islet tissue in all the cases.

## Discussion

Morphological and clinical studies have suggested that impaired glucose tolerance in CF and CFRD results from beta-cell dysfunction and insulin deficiency^16^. Observations from human CF pancreata of decreased islet density and relative proportion of islet beta-cells, and of islets embedded in strips of fibrosis formed the basis for the view that insulinopenia observed in patients with CF is caused mainly by beta-cell destruction secondary to chronic inflammation and pancreatic fibrosis^6–10,17–19^. It is intuitive that the chronic inflammatory environment contributes to beta-cell dysfunction and affects endocrine cell viability. However, the natural history of islet injury in CF and CFRD is unknown. The current morphologic evidence of reduced islet and beta-cell population in CF comes from studies in adolescent and young adult patients. The pancreata of these patients have been subjected to a chronic pathological process, and, therefore, the islet damage observed in these tissues may represent a more advanced stage of a long-standing process. On the other hand, impaired glucose tolerance is frequently observed in young children with CF, and CFRD, and rarely has also been diagnosed in children before two years of age^20–23^. To investigate whether islet changes begin in the CF pancreas in the immediate postnatal life and to what extent these changes are associated with the severity of pancreatic pathology, we undertook a morphological study of pancreatic tissues from neonates, infants and very young children with CF. To our knowledge, this is the first study of islets from such young children, which resulted in three notable findings.

First, we show that in children with CF under four years of age, the relative beta-cell areas and percentage of beta cells were reduced to about 50% of controls. The consistency in the insulin-positivity among the young CF tissues indicates that, at least during the first years of life, the islet and beta-cell numbers are not substantially reduced due to the severity of exocrine damage. These findings suggest that events other than those related to the ongoing inflammatory process in the surrounding exocrine pancreas contribute to a reduction in beta-cell population. It is possible that these events may have already taken place during developmental stages, and are characterized by deficiency in the growth of exocrine tissue^24,25^. It can be speculated that if pancreatic secretions are already altered in late pregnancy due to defective CFTR function in duct epithelial cells, then the accumulation of these secretions in pancreatic ducts may create an environment that hinders beta-cell expansion by affecting the survival of beta-cell progenitors or their fate specification towards beta-cells, or beta-cell proliferation^26,27^. While the number of beta cells per islet was reduced in the young CF pancreas, the islet areas and their size distribution in these tissues remained comparable to controls. This may be indicative of altered competence of endocrine progenitor cells towards generation of beta cells rather than a depleted pool of endocrine progenitors, and/or altered beta-cell proliferative capacity^27–30^. Interestingly, exposure of fetal rodent pancreas to the CFTR inhibitor Gly-H101 resulted in increased numbers of endocrine progenitor cells^31^. The mechanism behind this observation is not clear, and whether human fetal pancreatic cells would respond similarly to the chemical is not known.

Second, we show that proliferating insulin-positive cells were scarce or absent in young CF pancreata, while these cells were observed in all young control tissues^27^. Further, cells co-labeled for insulin and CK19, considered immature cells differentiating from the progenitor cells^14^, were present in the controls, but were rarely detected in young CF pancreata. The reasons for the defective beta-cell growth in the CF pancreas are not known. It may be that the already reduced beta-cell population at birth may not contain beta cells with proliferative capacity. Structural alterations, such as cellular atrophy and cystic dilation, affecting all the branches of the pancreatic duct system may lead to functional incapacitation of the endocrine progenitors located in duct epithelium or their physical loss, which will deplete the number of cells with capacity to generate new beta cells. Also, the inflammatory microenvironment and the pro-inflammatory mediators to which both pancreatic ducts and islets are exposed in chronic pancreatitis may create unfavorable conditions for beta-cell growth.

The third novel finding relates to the presence of inflammatory infiltrates in the CF islets. The inflammatory cells were dispersed and did not form aggregates which define classical insulitis in type 1 diabetes (T1D)^32^. Moreover, the patchy occurrence of islets with inflammatory infiltrates, characteristic of the T1D pancreas, was not evident in the CF tissues. These observations together with the lack of circulating islet cell autoantibodies are compatible with the view that beta-cell loss in CFRD is not due to an autoimmune process^33,34^. Although the islet cell infiltrates in CF were not extensive, the leukocyte interactions with islet cells and secretory products may affect beta-cell performance^35,36^. It is possible, therefore, that the presence of inflammatory cells juxtaposed to endocrine cells may further contribute to islet injury and dysfunction in CF. To what extent islet infiltrates contribute to islet damage in CF remains to be determined.

Our study clearly shows that islets are preserved in young CF pancreata. This finding, coupled with comparable pancreas weights reported in infants with and without CF^12,13^, suggests that early in postnatal life the tissue remodeling process going on in the extra-islet tissue does not lead to a change in the pancreas size. This is possible since inflamed tissues in the initial phases of inflammation are characterized by the presence of abundant extracellular matrix, edema, and inflammatory infiltrates^37^. Occurrence of islet-cell clusters closely associated with duct-like structures, likely formed as a result of acinar atrophy, may be indicative of a tissue remodeling process dictated by the loss of exocrine cells and their replacement by a more rigid fibrotic tissue. These duct-associated small endocrine cell clusters were not present in adult CF pancreata, suggesting that endocrine cells organized in fully developed islets survive better in an inflammatory environment than those occurring in smaller clusters.

Archival pancreas tissues from very young children with CF are very rare, therefore genotype-phenotype correlations at the level of beta-cell mass were difficult to establish. We did not observe a relationship between the severity of the CFTR mutation and the degree of reduction in beta-cell mass in the young CF tissues. Twelve of the 16 very young children with CF had severe genotypes, and the other four had meconium ileus, likely reflective of severe CFTR genotype. In all the young cases, the relative proportion of islet beta cells in their pancreas tissues varied from 29–47%, which was significantly lower compared to age-matched controls. To help account for the possibility that ethnicity might correlate with islet pathophysiology, Mestizo and non-Mestizo individuals were included in both young CF and control groups; no correlations with ethnicity were observed. These young CF tissues also showed different degrees of exocrine damage, varying from mild to severe. The three adult cases with CF were positive for the severe CFTR genotype, and their pancreata showed extensive loss of exocrine tissue. It is possible that mild CFTR mutations may lead to mild clinical manifestations and histopathologic changes. It is also possible that other factors than the CF gene defect itself can affect the chloride transport and preserve to some extent pancreatic function^38^.

All CFRD cases in our series had reduced islet density and lower islet cell number versus CF tissues, and some of them contained apoptotic beta-cells, which is compatible with loss of islet cells, and eventually loss of whole islets^6^. Interestingly, these tissues showed the lipoatrophic or “fatty” morphologic pattern, with islets occurring as single units surrounded by large areas of adipose tissues in the absence of fibrosis. This contrasts with islet distribution patterns in CF where islets were present in larger numbers, closely located to each other, and embedded in fibrosis. Differences in islet distribution and morphologic patterns that influence the pancreas volume^39,40^ may explain the variation in relative endocrine areas in the adult CF tissues. What determines the further development of chronic pancreatitis toward establishment of fibrosis or adipose tissue is not known. However, the data suggest that fat transformation of the pancreas is deleterious to islet cells in CF. Some of the CFRD cases had amyloid deposits in islets, while such deposits were not observed in the young CF islets. Thioflavin S identifies amyloid fibrils, however, it doesn’t detect pre-fibrillar amyloid monomers or oligomers^41–43^. Therefore, while the organized amyloid structures may not have formed in the young CF islets, the formation of pre-fibrillar amyloid species in these islets cannot be excluded.

The impact of chronic pancreatitis on beta-cell function and survival has been stressed by a recent study that suggested an association between exocrine pancreas deficiency and CFRD among CF patients with severe CFTR mutations^44^. Other studies have suggested that an intrinsic beta-cell defect is the major cause of beta-cell dysfunction in CF rather than beta-cell damage caused by pancreas fibrosis. These studies showed that patients with CF and with normal glucose tolerance exhibited a decrease in peak plasma insulin levels, and that abnormalities in insulin secretion were present in CF patients with preserved pancreas exocrine function^45,46^. In addition, gene variants associated with beta-cell dysfunction and type 2 diabetes are also associated with CFRD, suggesting that some of the same intrinsic beta-cell defects occur in CFRD^47^.

In conclusion, our study clearly establishes an altered cellular composition, early beta cell deficiency, and impaired growth capacity of beta-cells as properties for pancreatic islets in infants and young children with CF in the absence of CFRD. These changes exist independently of extent of exocrine damage and pancreatic fibrosis. Our observations are compatible with the view that impaired beta-cell growth in the young CF pancreas prevents the expansion of an already smaller -sized beta cell population present at birth, which results in an inadequate beta-cell mass incapable of meeting metabolic demands. Exposure to an inflammatory environment and the likely presence of additional intrinsic defects in islet endocrine cells may contribute to exacerbation of islet and beta-cell dysfunction, and development of CFRD. Our novel observations provide the morphologic evidence for the impaired beta-cell function observed in the pediatric population younger than four years and are to be taken into account in early screening for abnormal blood glucose in very young children with CF.

## Methods

### Human pancreas

Pancreatic tissues were archival autopsy pathological specimens or organ donors obtained through the JDRF Network for Pancreatic Organ Donors (case 6404, CFRD1; case 6136, CFRD3; case 6105, CFRD5; case 6398, CFRD6) or National Disease Research Interchange (CFRD4, CFRD8). Tissues were collected from 1990 to 2000, from Instituto Nacional de Pediatría and Hospital Infantil de Mexico Federico Gomez, Mexico City (CF1, CF2, CF5-CF7, CF9-CF16, N1, N2, N5-N11), Hautepierre Hospital, Strasbourg (CF3, CF4, N3, N4), and Vanderbilt University Medical Center, Nashville, TN (CF17-CF19, CFRD2, CFRD7). CF diagnosis was based on clinical features and testing for exocrine pancreas function, sweat test and molecular genetics, and histopathology. Clinical characteristics are shown in Table 2 and Supplementary Table S2. Tissue sections belonged to the pancreatic body. Normal tissues came from individuals who perished from non-pancreatic pathologies. Measured parameters between Latino and Caucasian samples showed no difference. There is no evidence of a phenotype or histopathology relationship with the patient ethnicity^48,49^. All the experiments with these de-identified archival autopsy tissues from deceased patients were carried out with the approval of the Institutional Review Boards of the Instituto Nacional de Pediatria and Hospital Infantil de Mexico Federico Gomez, Hautepierre Hospital, and Vanderbilt University Medical Center.

**Table 2:** Clinical characteristics of the study cases and tissue source.

Case	CF young	CF adult	CFRD	Control young	Control adult
Number	16	4	8	11	5
Age at death (yr)	0.8 ± 1.1	29 ± 9	31 ± 7	0.7 ± 1.1	32 ± 4
(range)	(5d to 4 yr.)	(22 to 43)	(21 to 44)	(1 m to 4 yr.)	(29 to 39)
Gender					
Female	7	1	6	5	2
Male	9	3	2	6	4
Race/Ethnicity					
Mestizo	13	0	1	9	0
Caucasian	3	4	7	2	6
Tissue source					
Autopsy	16	4	4	11	3
Donor	0	0	4	0	3

### Histologic evaluation of the exocrine pancreas pathology

To evaluate the severity of histopathological changes in the CF exocrine pancreas, we used a grading system (1 to 5) (Supplementary Table S3, Supplementary Fig. S10), based on the scoring system previously used for human chronic pancreatitis^50^. For completeness, quantification of the loss of chymotrypsin-positive (CHYM) acinar cells and the presence of ductal-like structures (dilated acini lined by atrophic acinar cells)^51^ were added. Fibrosis was evaluated as perilobular, intralobular and periacinar. Perilobular fibrosis was defined as fibrotic thickening of pancreatic interlobular septa. Intralobular fibrosis, as fibrotic strands that extended from the thickened interlobular septa into the pancreatic lobules and surrounded clusters of acini. Periacinar fibrosis was defined as fibrotic thickening of periacinar basement membranes. Loss of acinar parenchyma was assessed by the relative chymotrypsin-positive (CHYM) tissue area. Grade N (normal) was assigned to normal pancreas tissues without any detectable lesions and included: absence of fibrosis, acinar parenchyma occupying >95% of the pancreas tissue area, absence of duct-like structures, and absence of pancreatic duct dilation.

### CFTR mutation analysis

Genotyping for CFTR mutations was done via a multiplex assay to screen for 159 CFTR variants using open-platform matrix-assisted laser desorption/ionization time-of-flight (MALDI-TOF) mass spectrometry^52^. Samples were twice extracted with xylene to remove the paraffin, precipitated with ethanol, and digested using Proteinase K and Tween 20 (TE digestion buffer: Sigma cat. #T9285, Tween-20 Sigma cat. #P-9416, Proteinase K Qiagen cat. #19131). DNA was purified using the Qiagen QIAamp DNA Mini Kit (Valencia, CA).

### Immunohistochemistry

As previously described^53^, sections were incubated overnight with the primary antibodies listed in Supplementary Table S4. Sections were washed in PBS (pH 7.4), incubated with biotinylated- or fluorescent-labeled secondary antibodies for 60′ at RT, and counterstained with hematoxylin or DAPI to visualize the nuclei. The antibodies used in the study are routinely used in diagnostic islet pathology. They were validated on pancreas tissues from control patients who died of non-pancreatic pathologies. No differences were observed in immunostaining patterns for all antibodies used, between archival and organ donor tissues.

### Evaluation of islet cell composition and cell proliferation

Consecutive sections were co-stained for synaptophysin and either insulin, glucagon, somatostatin, or pancreatic polypeptide. The number of insulin-positive (INS), glucagon-positive (GLUC), somatostatin-positive (SOM), or pancreatic polypeptide-positive (PP) cells that co-stained with synaptophysin were counted, and the number of synaptophysin-positive (SYN) and INS cells per islet and the relative proportion of co-stained cells calculated. To ensure that all beta cells were detected, we stained two consecutive sections for either synaptophysin or a mixture of insulin, glucagon, somatostatin and PP antibodies, and counted the number of positive cells in the islets present in both sections. The number of SYN cells agreed with those stained for the four major islet hormones. In addition, CF and control tissues were co-labelled for insulin and proinsulin using an anti-human C-peptide/proinsulin antibody that does not cross-react with human insulin. All the C-peptide/proinsulin-positive cells co-stained with insulin (Supplementary Fig. 11).

Ki67 labeling index was used to assess islet cell proliferation. To determine the presence of endocrine cells formed through neogenesis from ductal cells^54,55^, sections were co-stained for the human pancreatic duct cell marker CK19 and one of the islet hormones. The percentage of INS, GLUC, SOM, or PP cells that co-stained for CK19 was then calculated.

Apoptosis was assessed by TUNEL stain (catalog# MS7110, Millipore) according to the manufacturer’s instructions.

Between 2000 and 6000 endocrine cells were counted in the young normal, young CF, and adult normal tissues. All the endocrine cells present in the adult CF/CFRD pancreas sections were counted. Samples were evaluated blindly and slides were coded and examined randomly.

### Morphometric quantitation

Islets were defined as groups of >20 endocrine cells^56–58^ and smaller groups were defined as clusters. Estimation of the relative pancreatic area occupied by SYN, INS, or CHYM cells was performed as described^53^, using a Nanozoomer Digital Pathology slide scanner (Hamamatsu; Bridgewater, New Jersey) at the Cellular and Molecular Imaging Core, DRC University of Washington, and the Visiopharm software (Hoersholm, Denmark).

### Evaluation of inflammatory cell infiltration

As previously described^53^, we determined (1) the percentage of islets with LCA (leukocyte common antigen)-, CD3-, or CD68-positive cells adjacent to endocrine cells, and (2) the number per islet of LCA-, CD3-, or CD68-positive cells in contact with endocrine cells. The density of LCA-, CD3-, or CD68-positive cells in the exocrine tissue was expressed as the number of LCA, CD3, and CD68 cells/mm^2^ tissue.

### Assessment of islet amyloid deposition

Sections were stained for insulin and thioflavin S^59^. Pancreas from human islet amyloid polypeptide transgenic mice, provided by Dr. Rebecca Hull at the UW, served as positive controls.

### Statistical analysis

Data were compared using the Mann-Whitney, Kruskal-Wallis, and the Spearman rank correlation tests. *P* values less than 0.05 were considered statistically significant.

## Electronic supplementary material

Supplementary infotmation
