# Supplementary material for: Structural abnormalities in islets from very young children with cystic fibrosis may contribute to cystic fibrosis-related diabetes
Source: Sci Rep. 2017 Dec 8;7:17231. doi: 10.1038/s41598-017-17404-z (PMC5722914; doi:10.1038/s41598-017-17404-z)
Supplement: Supplementary file 1 — Supplementary infotmation [file 41598_2017_17404_MOESM1_ESM.pdf]

ONLINE SUPPLEMENTARY INFORMATION

Structural abnormalities in islets from very young children with cystic fibrosis may contribute to cystic fibrosis-related diabetes.

Marika Bogdani, Scott M. Blackman, Cecilia Ridaura, Jean-Pierre Bellocq, Alvin C. Powers, and Lydia Aguilar-Bryan

Inventory of Supplementary Information

<u>*Supplementary Tables*</u>

- Supplementary Table 1. Quantitative analysis of pancreatic tissue changes.
- Supplementary Table 2. Clinical characteristics of study cases.
- Supplementary Table 3. Grading system of exocrine pancreas pathology in cystic fibrosis.
- Supplementary Table 4. Primary antibodies used for immunohistochemistry.

<u>*Supplementary Figures*</u>

- Supplementary Figure 1, Hematoxylin-eosin staining of normal, CF, and CFRD pancreas.
- Supplementary Figure 2, The grade of the severity of exocrine pancreas damage as a function of age.
- Supplementary Figure 3, Immunohistochemistry for chymotrypsin and synaptophysin in young normal and CF pancreas.
- Supplementary Figure 4, Immunohistochemistry for synaptophysin in adult normal, CF, and CFRD pancreas.
- Supplementary Figure 5, Immunohistochemistry for islet hormones in young normal and CF pancreas.
- Supplementary Figure 6, Quantitative analysis of islet area and cellular composition.
- Supplementary Figure 7, Immunohistochemistry for glucagon and CK19.
- Supplementary Figure 8, Distribution of inflammatory cells in islets and extra-islet tissue.
- Supplementary Figure 9, β-cell apoptosis.
- Supplementary Figure 10, Different degrees of pancreatic duct dilation in CF.
- Supplementary Figure 11, Immunohistochemistry for insulin and C-peptide/proinsulin.

Supplementary Table 1. Quantitative analysis of pancreatic tissue changes

Young CF

Case ID	Age	% pancreas tissue area CHYM (+)	% pancreas tissue area SYN (+)	% islet cells INS (+)	CF histopathologic grade
CF 1	5 d	10	3.5	33	4
CF 2	6 d	42	8.2	29	2
CF 3	10 d	85	5.2	46	1
CF 4	2 m	82	1.7	30	2
CF 5	3 m	83	2.7	40	1
CF 6	3 m	85	4.1	39	1
CF 7	4 m	61	1.2	36	2
CF 8	6 m	52	3.7	23	3
CF 9	6 m	59	2.1	44	4
CF10	6 m	62	3.0	33	3
CF11	7 m	60	1.1	33	3
CF12	7 m	50	2.3	37	3
CF13	1 yr	10	1.1	27	4
CF14	1.7 yr	29	5.2	47	3
CF15	2.7 yr	40	3.0	42	4
CF16	4 yr	5	1.7	47	5
		**51 ± 27[a,b]**	**3.1 ± 1.9[d]**	**37 ± 7[c]**	**3 ± 1[f]**

Adult CF

Case ID	Age	% pancreas tissue area CHYM (+)	% pancreas tissue area SYN (+)	% islet cells INS (+)	CF histopathologic grade
CF17	25 yr	<0.1	8.7	32	5
CF18	26 yr	<0.1	3.0	55	5
CF19	43 yr	<0.1	0.3	53	5
		**<0.1**	**4.0 ± 3.5**	**47 ± 10[e]**	**5 ± 0**

CFRD

Case ID	Age	% pancreas tissue area CHYM (+)	% pancreas tissue area SYN (+)	% islet cells INS (+)	CF histopathologic grade
CFRD1	21 yr	<0.1	<0.1	31	5
CFRD2	26 yr	<0.1	1.8	23	5
CFRD3	29 yr	<0.1	<0.1	34	5
CFRD4	30 yr	<0.1	<0.1	40	5
CFRD5	31 yr	<0.1	<0.1	45	5
CFRD6	33 yr	<0.1	<0.1	32	5
CFRD7	35 yr	<0.1	0.2	28	5
CFRD8	44 yr	<0.1	<0.1	35	5
		**<0.1[c]**	**0.3 ± 0.6[e]**	**33 ± 7[e]**	**5 ± 0**

Young controls

Case ID	Age	% pancreas tissue area CHYM (+)	% pancreas tissue area SYN (+)	% islet cells INS (+)	CF histopathologic grade
N 1	1 m	83	2.2	60	N
N 2	2 m	81	3.7	71	N
N 3	3 m	85	2.7	54	N
N 4	3 m	80	2.0	58	N
N 5	4 m	75	3.1	63	N
N 6	5 m	87	3.8	66	N
N 7	6 m	80	2.2	72	N
N 8	7 m	89	3.7	78	N
N 9	7 m	90	5.2	63	N
N10	11 m	92	1.0	62	N
N11	4 yr	94	0.8	76	N
		**85 ± 6**	**2.8 ± 1.3**	**66 ± 8**	

Adult controls

Case ID	Age	% pancreas tissue area CHYM (+)	% pancreas tissue area SYN (+)	% islet cells INS (+)	CF histopathologic grade
N12	29 yr	98	1.1	82	N
N13	30 yr	97	1.3	80	N
N14	30 yr	97	0.8	78	N
N15	30 yr	98	0.7	69	N
N16	32 yr	98	1.4	71	N
N17	39 yr	96	1.1	77	N
		**97 ± 1**	**1.1 ± 0.3**	**75 ± 4**[g]	

[a]$p < 0.001$, [c]$p < 0.0001$, [e]$p < 0.01$ vs. age-matched controls

[b]$p < 0.001$, [d]$p = 0.03$, [f]$p = 0.003$ young CF vs. adult CF and CFRD

[g]$p < 0.001$ adult normal vs. adult CF and CFRD

Note 1. Percent (%) tissue area positive for the indicated marker represents the relative pancreas area stained by the marker.

Note 2. There was a tendency for CFRD tissues to contain a smaller relative proportion of islet INS-positive cells than the CF tissues.

N, normal; CF, cystic fibrosis; CFRD, cystic fibrosis-realed diabetes; CHYM, chymotrypsin; INS, insulin; SYN, synaptophysin.

Supplementary Table 2. Clinical characteristics of the study cases

Young CF

Case ID	Age, Gender	Race/ Ethnicity	Genotype	Mutation severity[a]	Clinical and histopathologic findings
CF1	5 d, M	M	qns[b]		Sweat test (122 mmol/L), Meconium ileus; CF, pancreas, lung and intestine, mucoviscidosis
CF2	6 d, M	M	R764X / R764X	S / S	Meconium ileus; CF, pancreas, lung and intestine, mucoviscidosis
CF3	10 d, M	C	qns		Meconium ileus; CF, pancreas, mucoviscidosis
CF4	2 m, M	C	ni/ni		Meconium ileus; CF, pancreas, lung and intestine, mucoviscidosis
CF5	3 m, M	M	G1069R / ni	I / unknown	Meconium ileus; CF, pancreas, lung and intestine, mucoviscidosis
CF6	3 m, F	M	F508del / 1924del7	S / S	Dehydration; CF, pancreas, lung and intestine, mucoviscidosis
CF7	4 m, M	M	F508del / 1924del7	S / S	Bilateral pneumonia (P. aeruginosa); CF, pancreas, lung and intestine, mucoviscidosis
CF8	6 m, F	C	F508del / G85E	S / S	Meconium ileus; CF, pancreas, lung and intestine, mucoviscidosis
CF9	6 m, M	M	F508del / F508del	S / S	Meconium ileus; CF, pancreas, lung and intestine, mucoviscidosis
CF10	6 m, F	M	L558S / R1066C	S / S	Bilateral pneumonia (P. aeruginosa); CF, pancreas, lung and intestine, mucoviscidosis
CF11	7 m, M	M	F508del / F508del	S / S	Meconium ileus; CF, pancreas, lung and intestine, mucoviscidosis
CF12	7 m, F	M	F508del / F508del	S / S	Meconium ileus; CF, pancreas, lung and intestine, mucoviscidosis
CF13	1 yr, M	M	G542X / ni	S / unknown	Sweat test (114 mmol/L); CF, pancreas and lung, mucoviscidosis
CF14	1.7 yr, F	M	2143delT / A559V	S / unknown	Bilateral pneumonia (P. aeruginosa); CF, pancreas, salivary glands, lung, mucoviscidosis
CF15	2.7 yr, F	M	F508del / 2055del9>A	S / S	Bilateral pneumonia (P. aeruginosa); CF, pancreas, lung, mucoviscidosis
CF16	4 yr, F	M	F508del / 711+1G>T	S / S	Bilateral pneumonia (P. aeruginosa), Pancreatic insufficiency; CF, pancreas and lung, mucoviscidosis

Adult CF

Case ID	Age, Gender	Race/ Ethnicity	Genotype	Mutation severity[a]	Clinical and histopathologic findings
CF17	25 yr, M	C	F508del / F508del	S / S	Pancreatic insufficiency; CF
CF18	26 yr, M	C	F508del / G551D	S / S	Pancreatic insufficiency, steroid-induced hyperglycemia; CF
CF19	43 yr, M	C	F508del / F508del	S / S	Pancreatic insufficiency, medication-induced hyperglycemia; CF

CFRD

Case ID	Age, Gender	Race/ Ethnicity	Genotype	Mutation severity[a]	Clinical and histopathologic findings
CFRD1	21 yr, F	M	F508del / F508del	S / S	CFRD for 5 years;<br>CF, pancreas weight 34 gr
CFRD2	26 yr, F	C	F508del / F508del	S / S	CFRD for 7 years;<br>CF
CFRd3	29 yr, F	C	F508del / K710X	S / S	CFRD for 3 months;<br>CF, pancreas weight 134 gr
CFRD4	30 yr, M	C	F508del / F508del	S / S	not reported,<br>CF
CFRD5	31 yr, F	C	F508del / F508del	S / S	CFRD for 3 months;<br>CF, pancreas weight 105 gr
CFRD6	33 yr, M	C	F508del / F508del	S / S	CFRD for 2 years;<br>CF, pancreas weight 207 gr
CFRD7	35 yr, F	C	F508del / F508del	S / S	CFRD for 1.5 years;<br>CF
CFRD8	44 yr, F	C	F508del / F508del	S / S	not reported;<br>CF

[a]Severe (S): ≥75% PI in CFTR2 (27); Intermediate (I): 50-75%; Mild (M): <50% PI.
[b]qns, insufficient DNA for CFTR genotype testing
ni, no CF-causing mutation on the panel was identified
M, Mestizo; C, Caucasian

Young controls

Case ID	Age, Gender	Race/ Ethnicity
N1	1 m, F	M
N2	2 m, F	M
N3	3 m, M	C
N4	3 m, M	C
N5	4 m, M	M
N6	5 m, M	M
N7	6 m, F	M
N8	7 m, F	M
N9	7 m, M	M
N10	11 m, M	M
N11	4 yr, F	M

Adult controls

Case ID	Age, Gender	Race/ Ethnicity
N12	29 yr, M	C
N13	30 yr, M	C
N14	30yr, M	C
N15	30 yr, M	C
N16	32 yr, F	C
N17	39 yr, F	C

Supplementary Table 3. Grading system of exocrine pancreas pathology in cystic fibrosis

Grade	Histopathological changes
N	No histopathological changes / no alterations in tissue morphology / no morphologic changes
1	Fibrosis: perilobular fibrosis only with minimal intralobular or periacinar involvement
	Acinar tissue changes: percent tissue area CHYM+, 80-95%; acini slightly dilated, no formation of duct-like structures
	Pancreatic duct dilation, mild: duct lumen becomes visible and lined by columnar epithelial cells
2	Fibrosis in 10-30% of tissue area
	Acinar tissue changes: percent tissue area CHYM+, 60-80%; duct-like structures present
	Pancreatic duct dilation, moderate: ducts lined by low cuboidal epithelial cells
3	Fibrosis in 30-60% of tissue area
	Acinar tissue changes: percent tissue area CHYM+, 20-60%; duct-like structures present
	Pancreatic duct dilation, marked: duct lined by flat epithelial cells
4	Fibrosis in 60-90% of tissue area
	Acinar tissue changes: percent tissue area CHYM+, 5-20%; duct-like structures present.
	Pancreatic duct dilation, cystic: ducts devoid of epithelial cells
5	Fibrotic or fat replacement of entire exocrine tissue
	Pancreatic duct dilation, cystic: ducts devoid of epithelial cells

N, normal pancreas histology
CHYM, chymotrypsin

Grade N (normal) was assigned to the normal pancreas tissues without any detectable lesions and includes: absence of fibrosis, acinar parenchyma occupying more than 95% of the pancreas tissue area, absence of duct-like structures, and absence of pancreatic duct dilation.

Fibrosis is evaluated as perilobular, intralobular and periacinar fibrosis.
Perilobular fibrosis was defined as fibrotic thickening of pancreatic interlobular septa.
Intralobular fibrosis was defined as fibrotic strands that extend from the thickened interlobular septa into the pancreatic lobules and surround clusters of acini.
Periacinar fibrosis was defined as fibrotic thickening of periacinar basement membranes.

The duct-like structures represent dilated acini lined by chymotrypsin-positive acinar cells and/or chymotrypsin-negative epithelial cells.

Loss of the acinar parenchyma was assessed by the relative chymotrypsin-positive (CHYM) tissue area.

Supplementary Table 4. Primary antibodies used for immunohistochemistry

Antibody	Catalog number	Source	Dilution
Chymotrypsin	ab155400	Abcam	1:50
Synaptophysin	ab8049	Abcam	1:200
Insulin	A18-0067	Invitrogen	1:1000
Glucagon	ab8055	Abcam	1:500
Somatostatin	ab2366	Abcam	1:200
Pancreatic polypeptide	ab113694	Invitrogen	1:200
C-peptide/proinsulin	ab1973	Abcam	1:2000
Ki67	ab15580	Abcam	1:100
CK19	ab15463	Abcam	1:50
LCA	GA751	DAKO	1:200
CD3	A0452	DAKO	1:100
CD68	M0814	DAKO	1:100

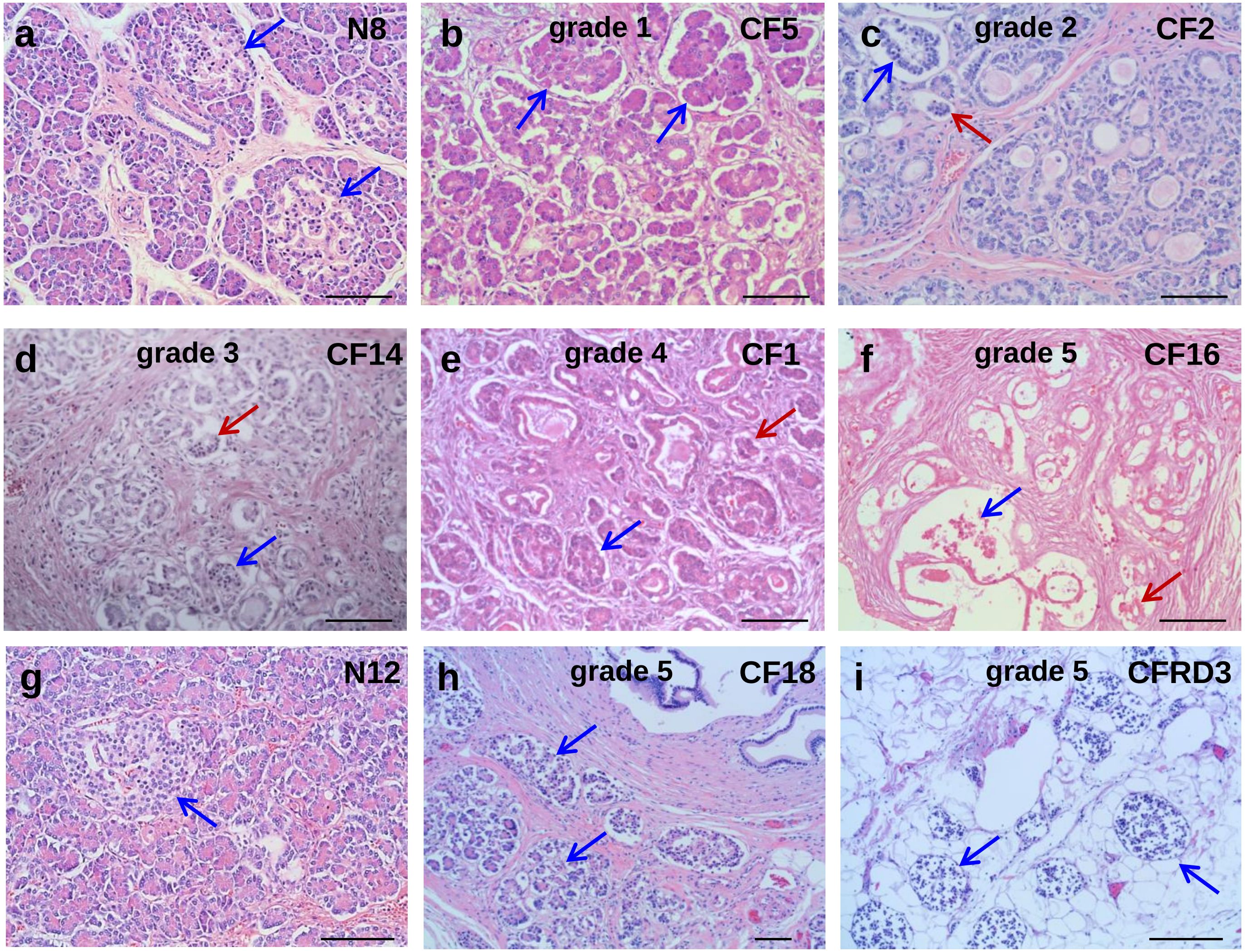

Supplementary Figure 1. Hematoxylin-eosin staining of young normal (**a**), young CF (**b, c, e, f**), adult normal (**g**), adult CF (**h**), and CFRD (**i**) pancreas. The CF pancreata show different degree of histopathological changes in the exocrine tissue. Blue and red arrows point to islets and endocrine cell clusters, respectively. Scale bars, 50 µm. In all adult CF and CFRD pancreas tissues, acinar tissue was entirely replaced by fibrosis or adipose tissue (**h**, **i**).

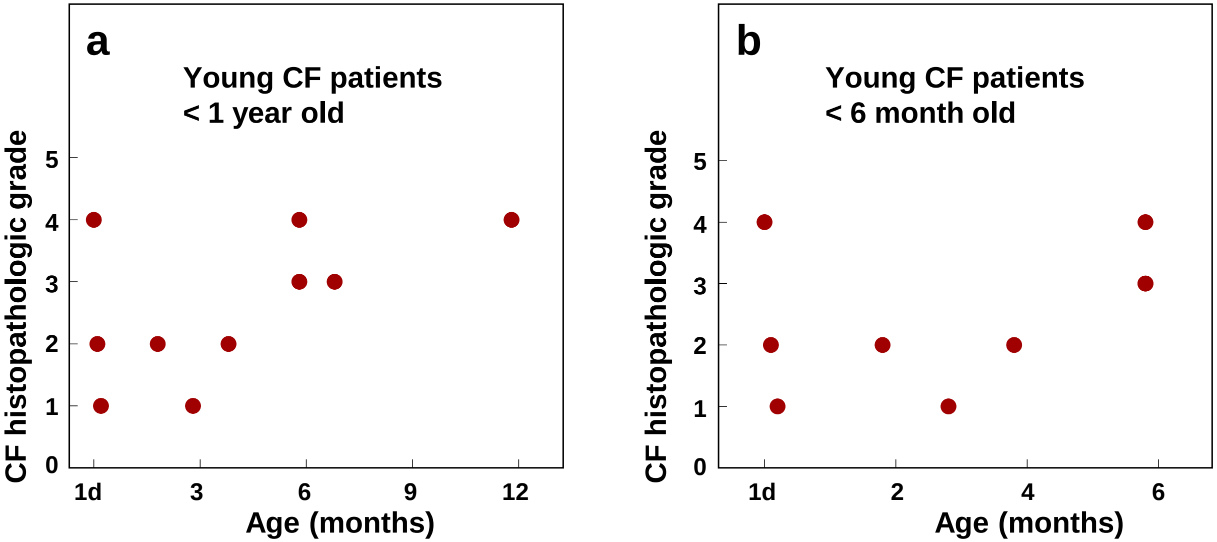

Supplementary Figure 2. Scatter plot of the grade of the severity of exocrine pancreas damage as a function of age. The correlation coefficient is $R^2 = 0.30$ for (**a**) and $R^2 = 0.12$ for (**b**).

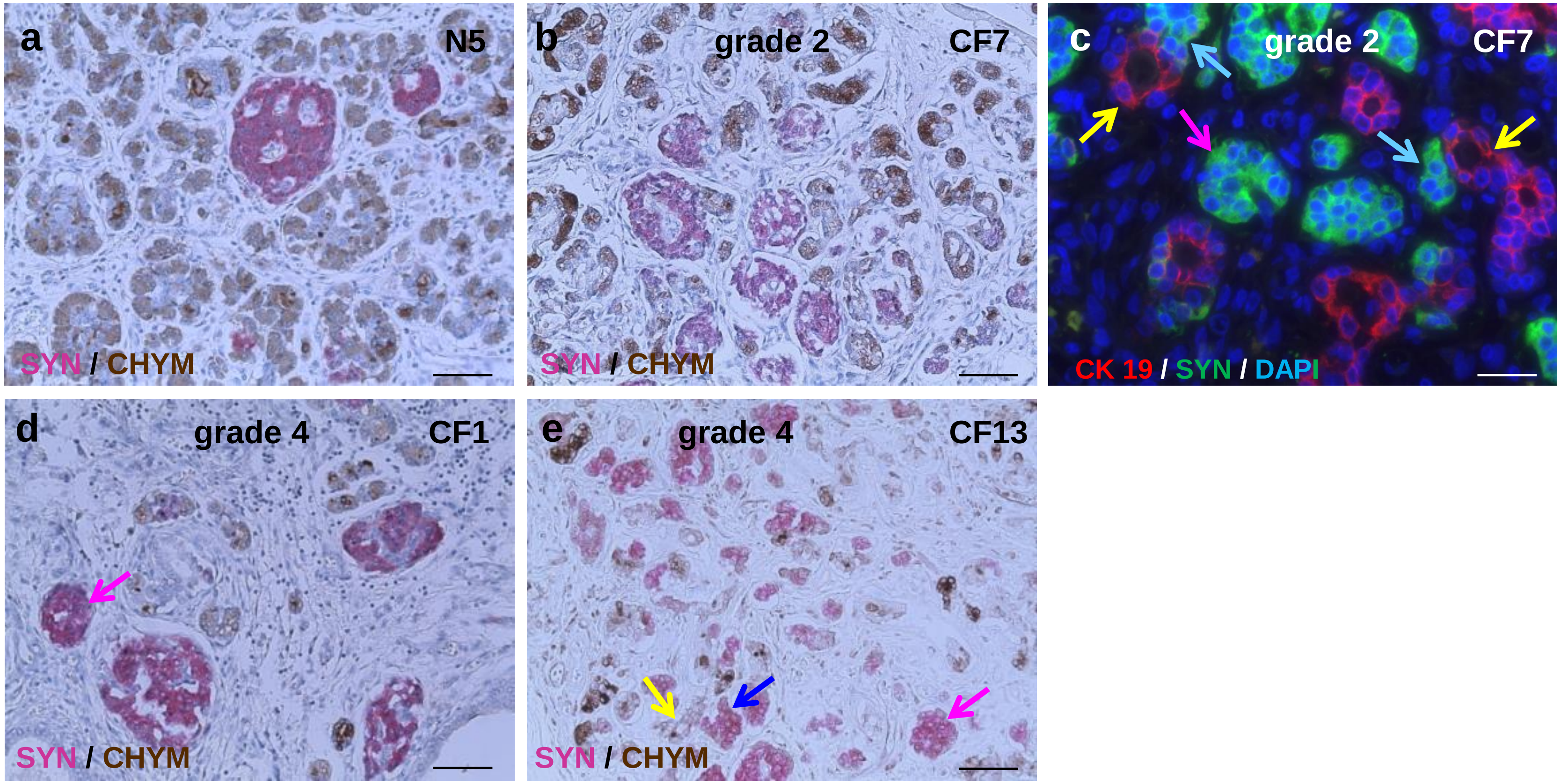

Supplementary Figure 3. Immunohistochemistry (IHC) for chymotrypsin (CHYM, brown) and synaptophysin (SYN, red) in young normal (**a**) and CF (**b, d, e**) pancreas. (**c**) IHC for SYN (green) and the pancreatic duct cell marker CK 19 (red). Dark and light blue arrows point to SYN-positive cells located adjacent to CHYM-negative duct or duct-like structures (yellow arrows), magenta arrows point to SYN-positive cells surrounded by fibrosis. Scale bars: 50 μm (**a, b, d, e**) and 25 μm (**c**).

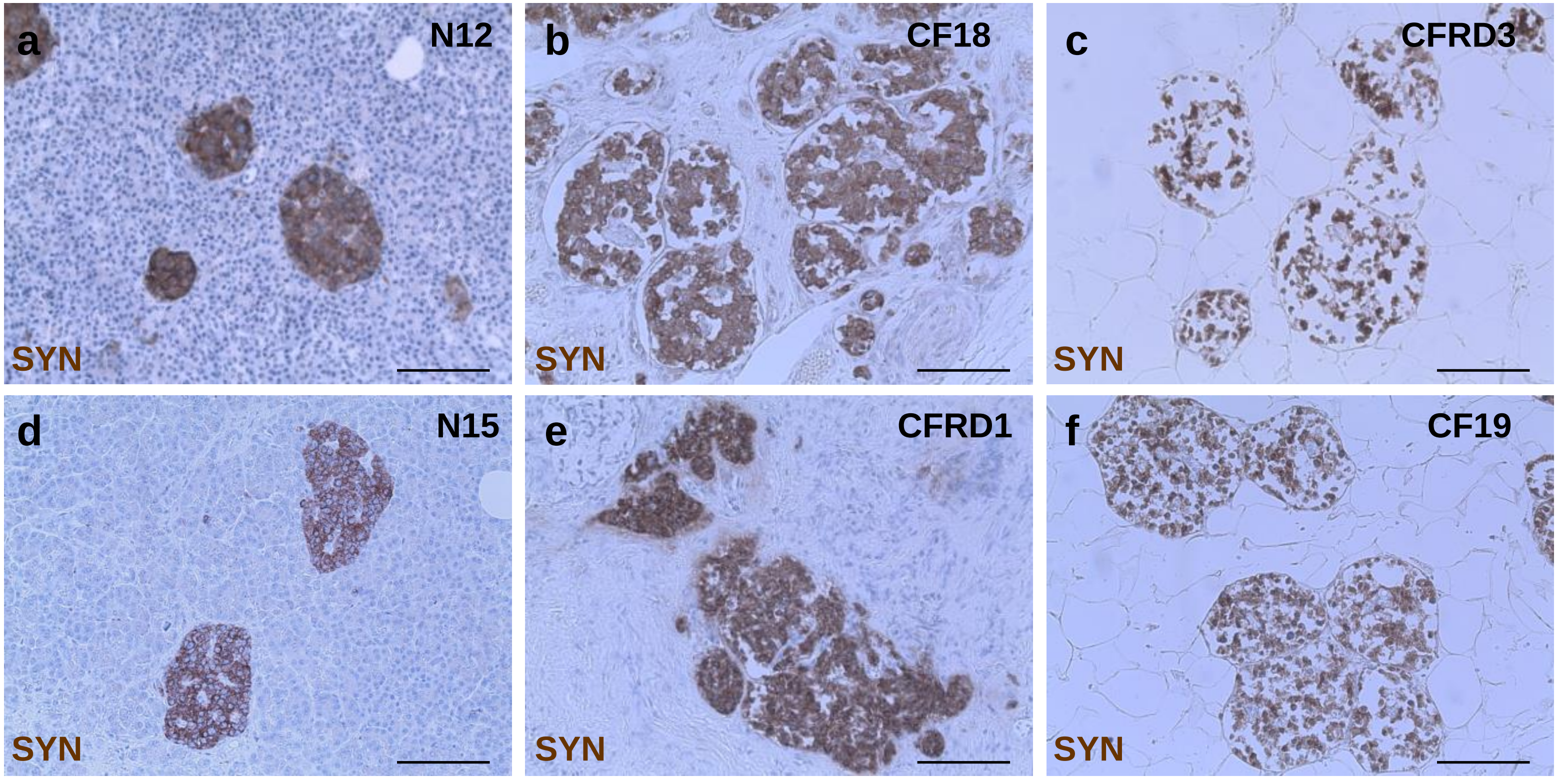

Supplementary Figure 4. Immunohistochemistry for synaptophysin (SYN) of adult normal (**a** and **d**), adult CF (**b** and **f**), and CFRD (**c** and **e**) pancreas. The adult CF and CFRD pancreas tissues showed either the "fibrotic" (**b** and **e**) or "lipoatrophic" (**c** and **f**) pattern. Scale bars: 100 µm.

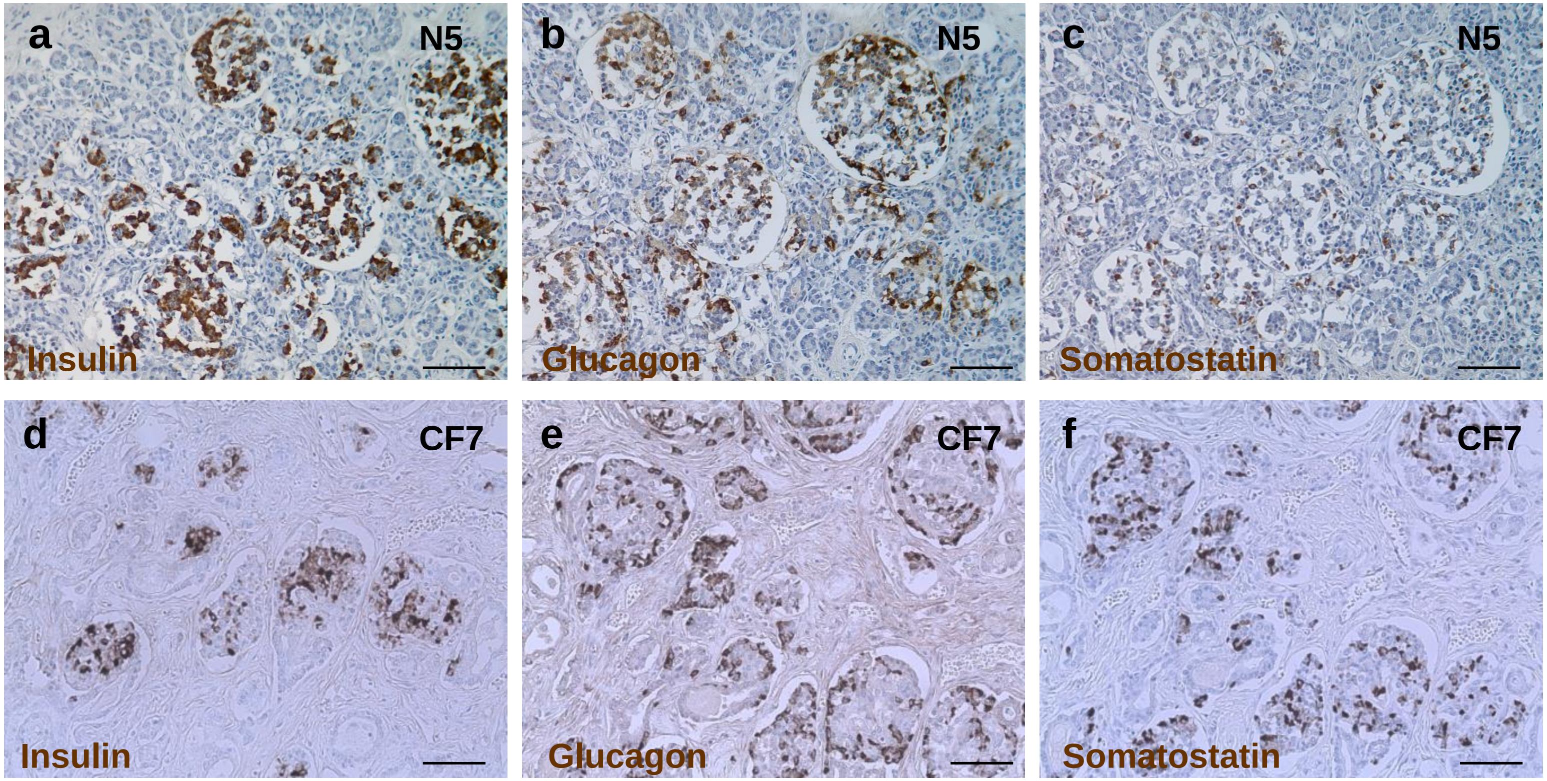

Supplementary Figure 5. Immunohistochemistry for insulin (**a** and **d**), glucagon **(b** and **e**), and somatostatin (**c** and **f**) in young normal (**a** - **c**) and CF (**d** - **f**) pancreas. Fewer insulin-positive cells and more glucagon-, and somatostatin-positive cells are present in the CF islets compared to normal islets. Scale bars: 100 µm.

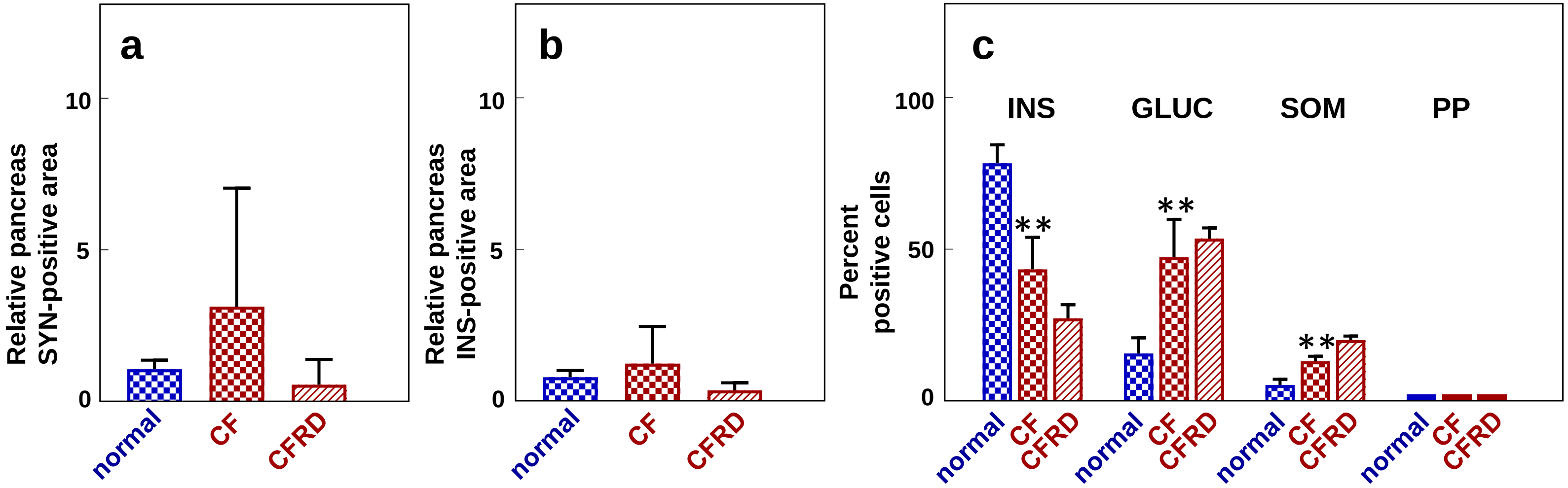

Supplementary Figure 6. Quantitative analysis of the relative SYN-positive (**a**) and INS-positive (**b**) areas, and islet cellular composition (**c**) in adult CF and CFRD pancreas tissues. SYN, synaptophysin; INS, insulin; GLUC, glucagon; SOM, somatostatin; PP, pancreatic polypeptide. Data are mean ± SD of measurements obtained from adult normal tissues (n = 6), adult CF (n = 3) and CFRD (n = 8) tissues. **$p < 0.001$ vs. normal tissues.

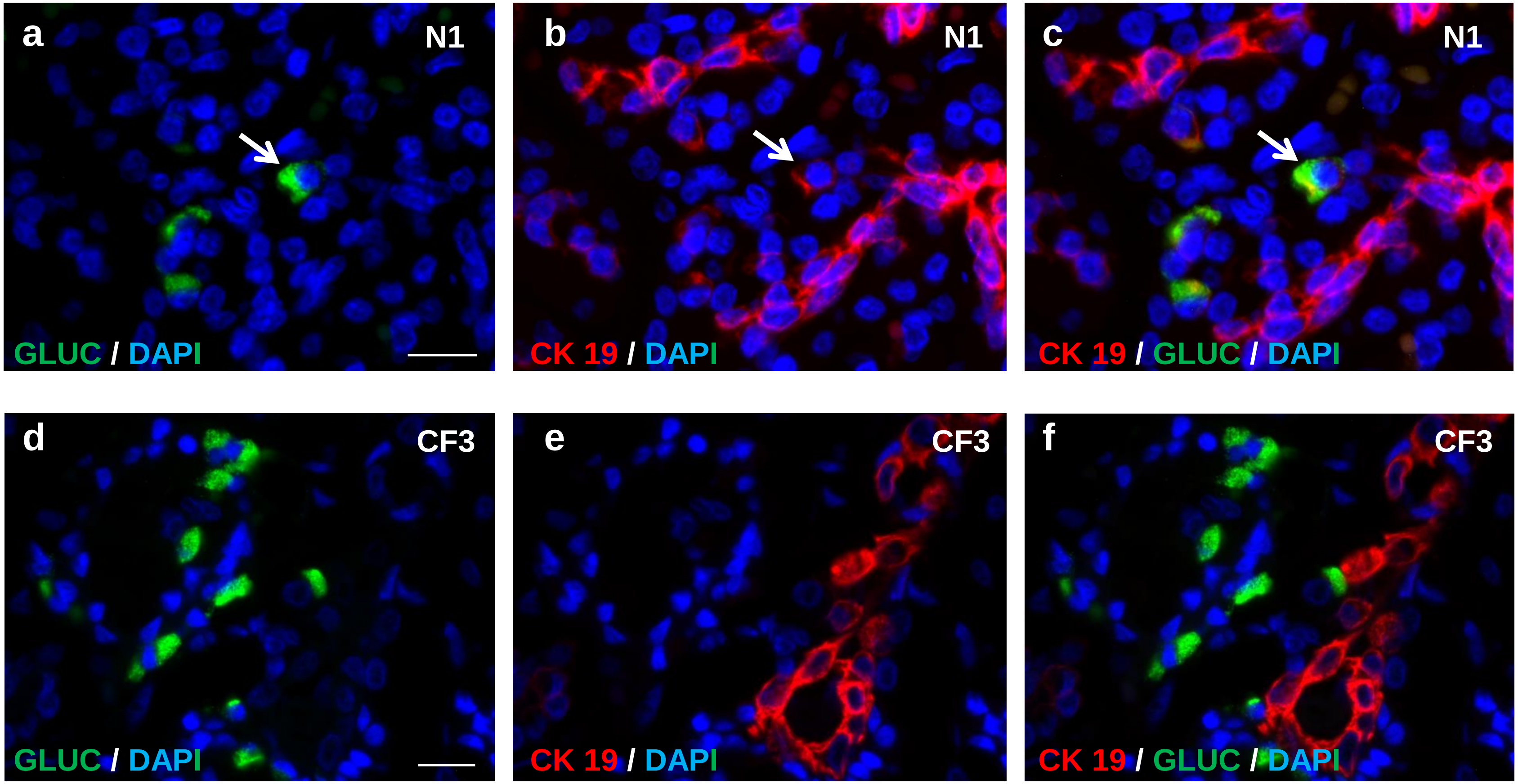

Supplementary Figure 7. Immunohistochemistry for glucagon (GLUC, green) and the pancreatic duct cell marker CK 19 (red) in pancreas tissues from a young control (**a** – **c**) and a young CF case (**d** – **f**). Nuclei are visualized with DAPI staining. White arrows point to a glucagon and CK19 double-positive cell. Scale bars: 25 µm.

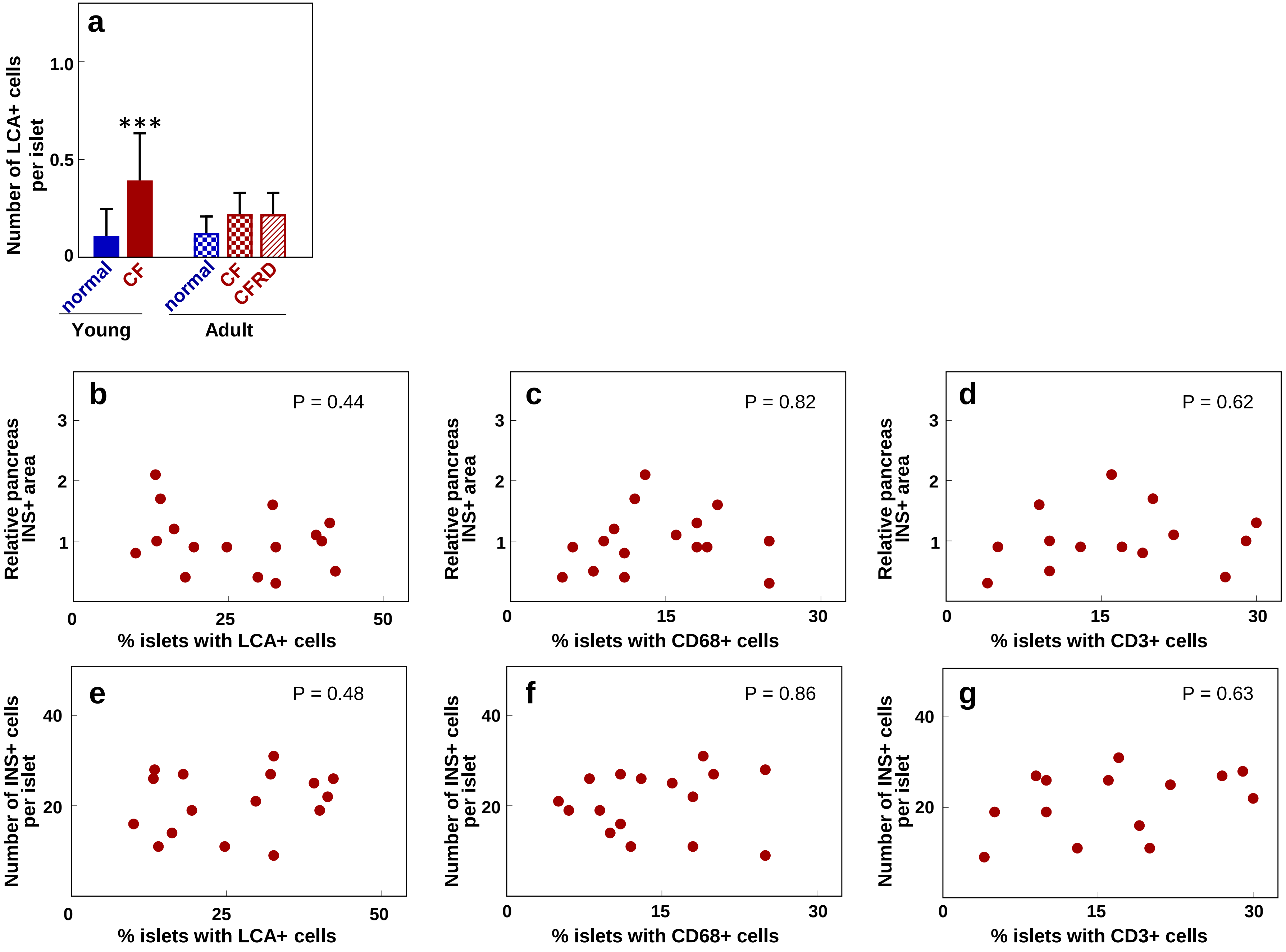

Supplementary Figure 8.

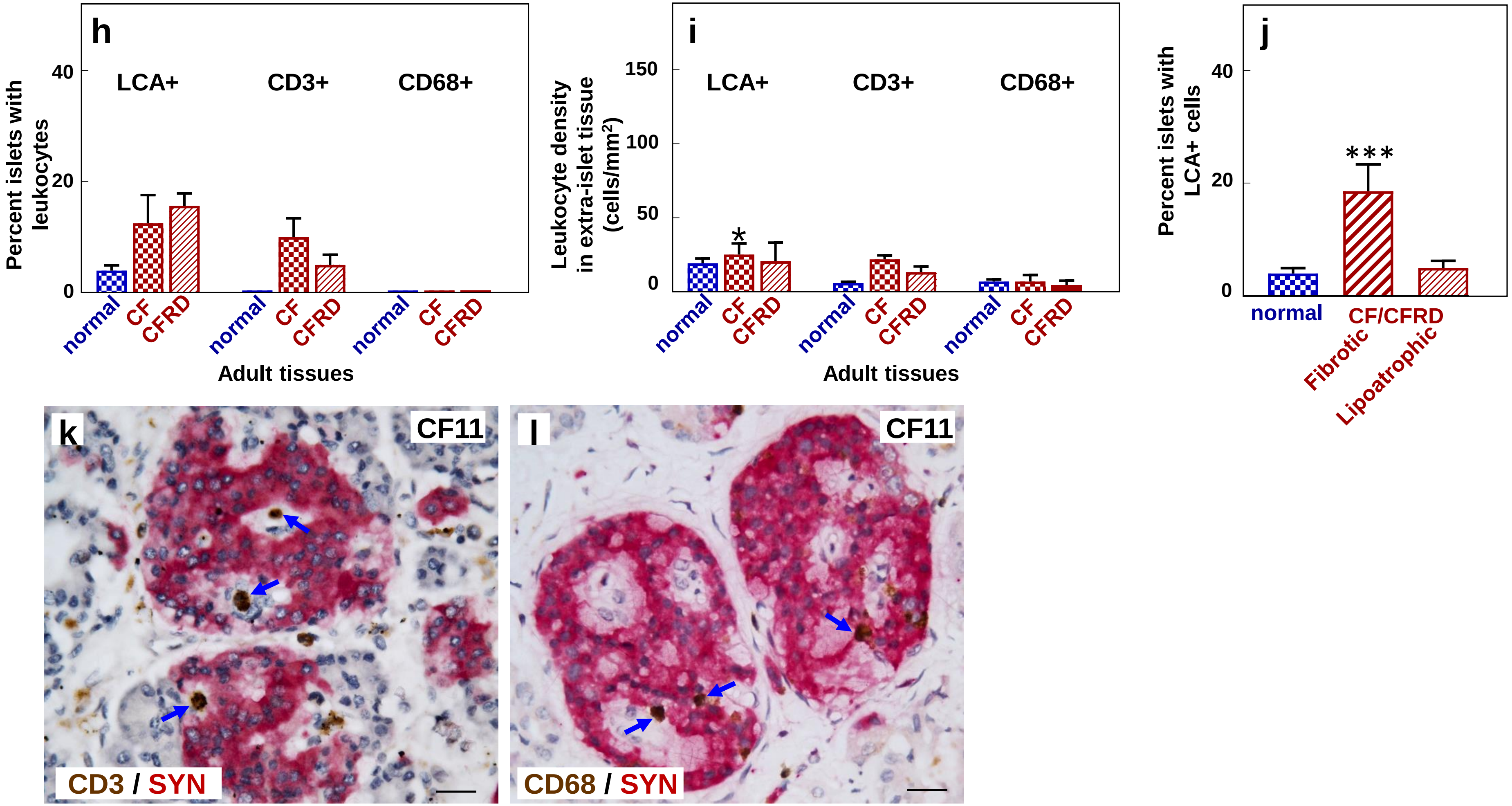

Supplementary Figure 8. **(a)** Quantitative analysis of the distribution of LCA+ inflammatory cells in islets of CF and control cases. (**b - d**) Scatter plot of relative pancreas INS+ areas as a function of the proportion of islets with inflammatory cells. (**e - g**) Scatter plot of islet INS+ cell number as a function of the proportion of islets with inflammatory cells. *P* values are indicated. (**h** and **i**) Quantitative analysis of the distribution of inflammatory cells in islets and extra-islet tissue in adult control and CF cases. $*p < 0.01$ vs. normal tissues. (**j**) Prevalence of pancreatic islets with LCA+ inflammatory cell infiltrates in CF tissues with "fibrotic" or "lipoatrophic" patterns. Data are mean ± SD of measurements obtained from adult normal tissues (n = 6), adult CF and CFRD tissues with the "fibrotic" pattern (n = 3), and adult CF and CFRD tissues with the "lipoatrophic" pattern (n = 8). $***p < 0.0001$ vs. normal tissues. (**k** and **l**) Immunohistochemistry for synaptophysin (SYN) in red and either CD3 (**k**) or CD68 (**l**) in brown. The arrows point to intra-islet inflammatory cells. Scale bars: 25µm.

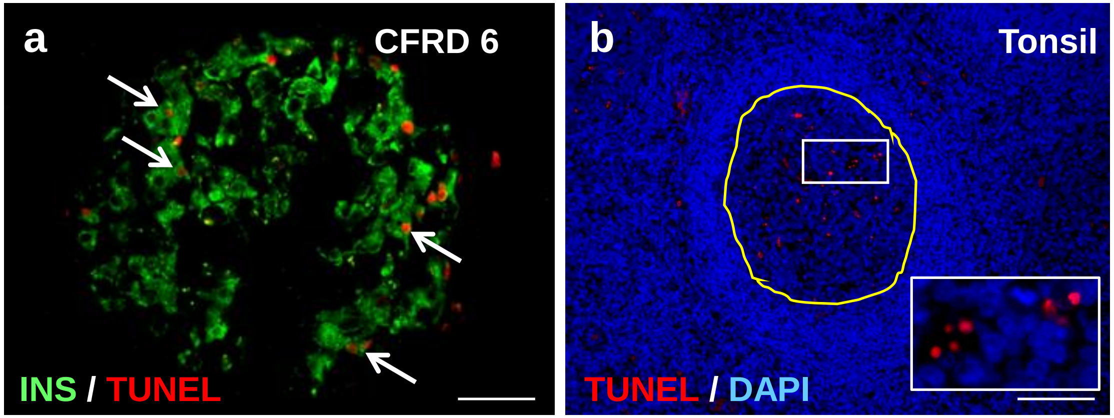

Supplementary Figure 9. β-cell apoptosis. TUNEL staining in a pancreatic islet from a patient with CFRD (**a**) and in a normal human tonsil (**b**). Arrows in (**a**) point to TUNEL-positive INS+ cells. Normal tonsil tissue from a healthy subject was used as positive control tissue for the TUNEL staining. Cells with TUNEL-positive nuclei are present in the follicular germinal center, which is delineated by the yellow line. The nuclei are visualized by DAPI. INS, insulin. Scale bars: 50 µm (**a**) and 100 µm (**b**).

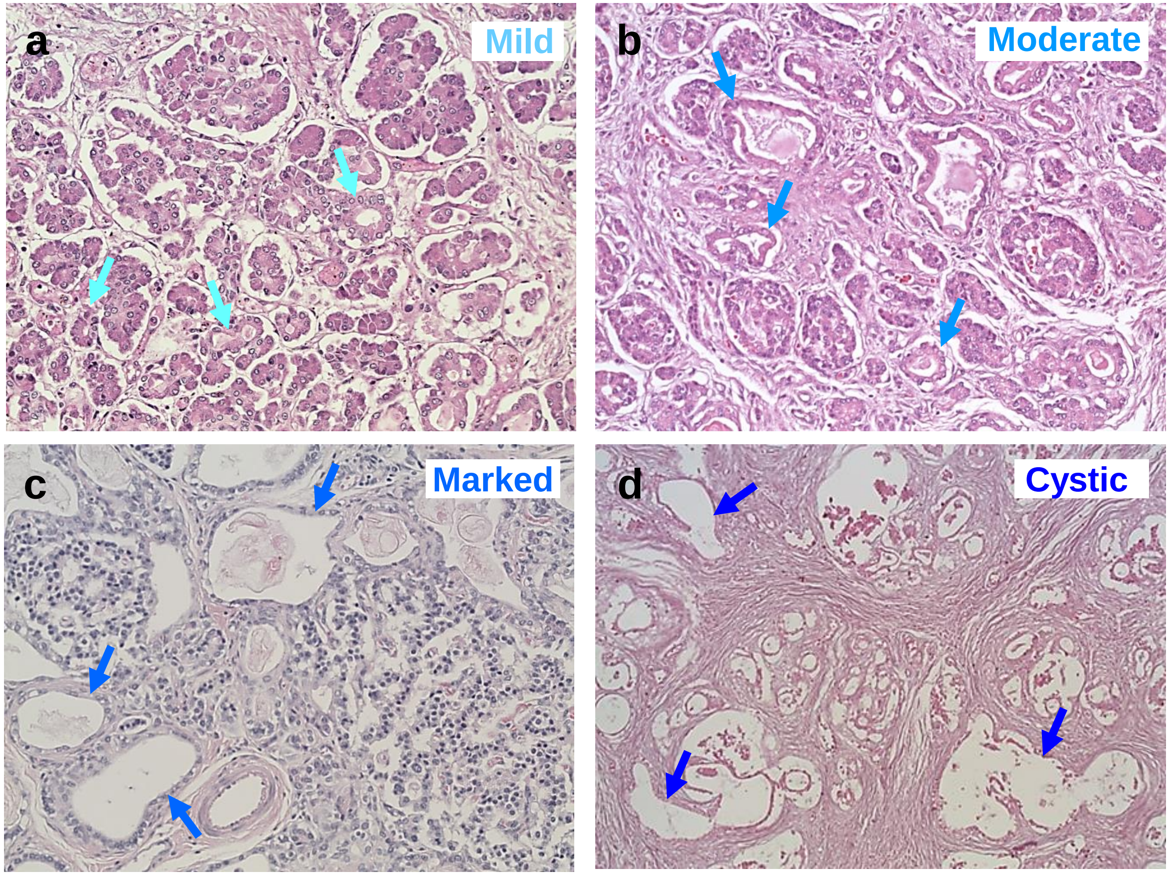

Supplementary Figure 10. Different degrees of pancreatic duct dilation in CF.
Hematoxylin-eosin staining of young CF pancreas showing different degree of pancreatic duct dilation.
(**a**) Pancreatic duct dilation, mild: duct lumen becomes visible and are lined by columnar epithelial cells.
(**b**) Pancreatic duct dilation, moderate: ducts are lined by low cuboidal epithelial cells.
(**c**) Pancreatic duct dilation, marked: duct are lined by flat epithelial cells.
(**d**) Pancreatic duct dilation, cystic: ducts are devoid of epithelial cells
Arrows point to pancreatic ducts.

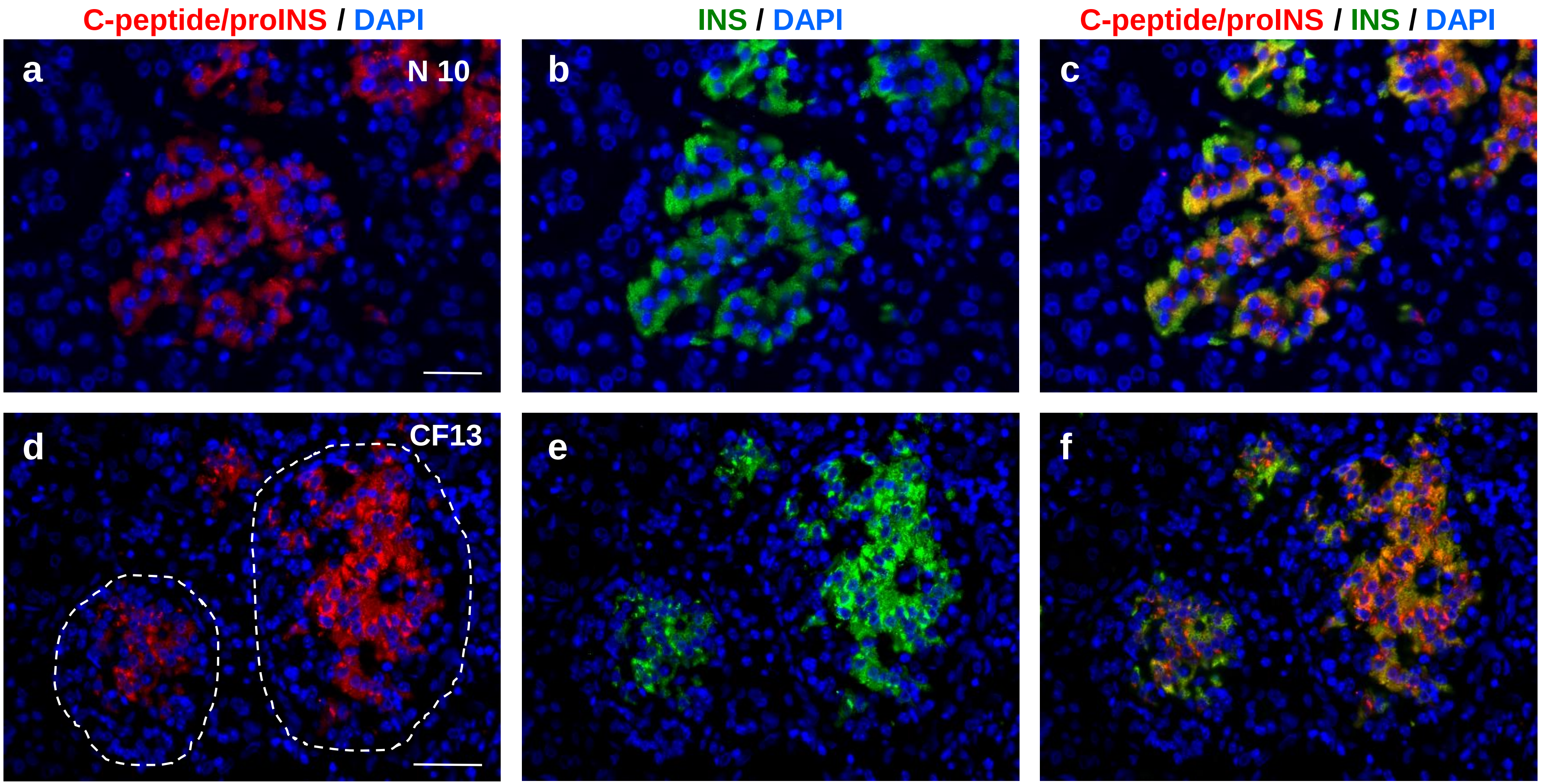

Supplementary Figure 11. Co-labeling for C-peptide/proinsulin in red and insulin (INS) in green in tissues from a young control (**a** – **c**) and a young CF (**d** – **f**) cases. The anti human C-peptide antibody recognizes the free C-peptide, located in the cytoplasmic secretory granules, and pro-insulin (proINS), the precursor of insulin which localizes in the endoplasmic reticulum and Golgi apparatus located near the cell nucleus. The dashed lines in (**d**) delineate the islet borders. Nuclei are visualized by DAPI staining. Scale bars: 50 µm.
